# Mining for Potent Inhibitors through Artificial Intelligence and Physics: A Unified Methodology for Ligand Based and Structure Based Drug Design

**DOI:** 10.1021/acs.jcim.4c00634

**Published:** 2024-06-06

**Authors:** Jie Li, Oufan Zhang, Kunyang Sun, Yingze Wang, Xingyi Guan, Dorian Bagni, Mojtaba Haghighatlari, Fiona L. Kearns, Conor Parks, Rommie E. Amaro, Teresa Head-Gordon

**Affiliations:** † Pitzer Center for Theoretical Chemistry, Department of Chemistry, University of California, Berkeley, California 94720, United States; ‡ Department of Chemistry and Biochemistry, University of California, San Diego, La Jolla, California 92093, United States; ¶ Departments of Bioengineering and Chemical and Biomolecular Engineering, University of California, Berkeley, California 94720, United States

## Abstract

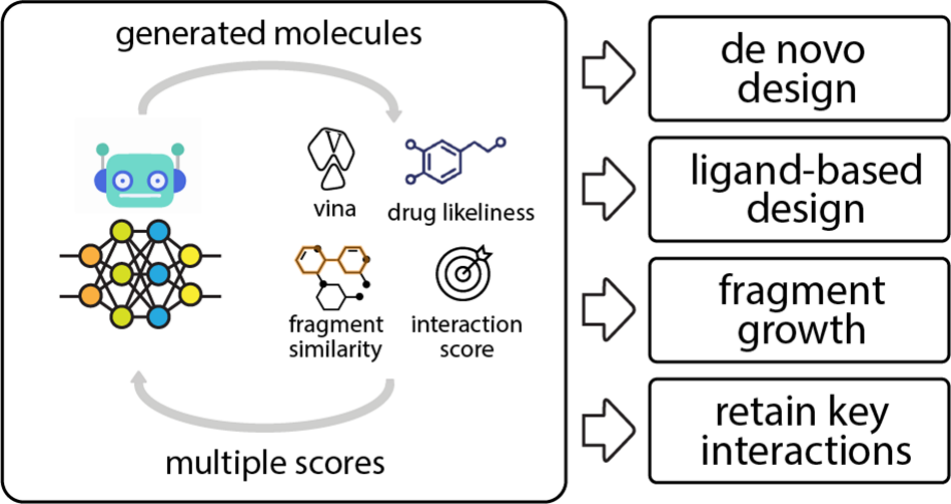

Determining the viability of a new drug molecule is a time- and resource-intensive task that makes computer-aided assessments a vital approach to rapid drug discovery. Here we develop a machine learning algorithm, iMiner, that generates novel inhibitor molecules for target proteins by combining deep reinforcement learning with real-time 3D molecular docking using AutoDock Vina, thereby simultaneously creating chemical novelty while constraining molecules for shape and molecular compatibility with target active sites. Moreover, through the use of various types of reward functions, we have introduced novelty in generative tasks for new molecules such as chemical similarity to a target ligand, molecules grown from known protein bound fragments, and creation of molecules that enforce interactions with target residues in the protein active site. The iMiner algorithm is embedded in a composite workflow that filters out Pan-assay interference compounds, Lipinski rule violations, uncommon structures in medicinal chemistry, and poor synthetic accessibility with options for cross-validation against other docking scoring functions and automation of a molecular dynamics simulation to measure pose stability. We also allow users to define a set of rules for the structures they would like to exclude during the training process and postfiltering steps. Because our approach relies only on the structure of the target protein, iMiner can be easily adapted for the future development of other inhibitors or small molecule therapeutics of any target protein.

## Introduction

Discovery of new drugs that inhibit target proteins usually follows either a screening-based approach or a rational design approach.^1,2^ If no explicit information about either the structure of the protein target or a ligand that might bind to the protein is available, the screening of a large database might be the only realistic approach to identify a starting point for drug development. However, any additional information about the structure of either the protein or the ligand can narrow down the search space significantly and encourages rational ligand based or structure based drug design.^2,3^ The process of rational drug design requires either exquisite domain knowledge and devoted time by an experienced medicinal chemist or using an automated workflow that relies on virtual screening of protein–ligand databases combined with physics-based modeling, such as docking simulations, to identify candidate molecules that may potentially bind to the protein target of interest.^4^

In order to identify promising small molecule therapeutics, existing high-throughput virtual screening approaches often evaluate comprehensive drug databases such as ChEMBL,^5^ PubChem,^6^ and ZINC.^7^ Even with the recent advent of the Enamine REAL library of 6–11 billion molecules,^8^ it is still dwarfed by estimates for the total number of possible synthesizable small molecules that range from 10^24^–10^60^.^9^ Unfortunately, due to the size of such established or expanded databases, screening all compounds according to sufficiently sophisticated structure based methodologies, such as flexible ligand docking, can be intractable. Even with simpler methods such as pharmacophore modeling or rigid body docking, it can still be time-consuming to navigate through the chemically feasible space, with a tendency toward false-positives being ruled in while false-negatives, i.e., potential optimum lead molecules, can be ruled out.^10,11^

With the rise of modern machine learning, a more clever exploration of the chemical space for drug-like molecules becomes possible, thanks to the development of generative models that can generate molecules represented as either strings or graphs.^12−22^ More interestingly, the distribution can be skewed toward molecules with specific properties such as drug likeness using techniques such as variational autoencoders (VAEs),^13,14^ transfer learning,^15^ and reinforcement learning (RL).^16−22^ However, early deep learning methods rely on one-dimensional (1D) sequence or two-dimensional (2D) chemical representations of the drug and protein and do not take full advantage of three-dimensional (3D) structural information on the putative drug, thereby constraining the ability to *generate* drugs with shape and molecular compatibility with the target active site. When SMILES-based generative models were combined with additional information about protein structure, ligand structure, or protein–ligand interactions, the quality of generated molecules improved in terms of predicted binding affinity or binding mode similarity with a reference binder.^23,24^ Recent work has explored chemical space in the vicinity of some starting molecular scaffold, running docking simulations on these derived molecules,^25^ but the chemical space that can be explored by such a method is still rather limited. Generation of atomistic structures of new compounds conditioned on the 3D structure of a provided pocket or constrained on interaction fingerprints has also been proposed with sequential growth of atoms,^26,27^ hierarchical buildup of 3D coordinates^28,29^ or generating all-atom coordinates at once using diffusion models.^30,31^ However, these existing methods have either been tailored toward *de novo* generation or a local optimization but lack the flexibility to extend from *de novo* design to structure or ligand based rational design. These additional capabilities are essential for continuously improving the binding potency between ligands and proteins after a hit molecule is already identified.

In this work, we propose a novel composite workflow, dubbed “iMiner”, that mines chemical space for new tight binding inhibitors by combining deep RL with real-time flexible ligand docking against a protein binding site. We represent putative inhibitors as Self-Referencing Embedded Strings (SELFIES)^32^ that are generated from an Average Stochastic Gradient Descent Weigh-Dropped Long Short-Term Memory (AWD-LSTM)^33^ recurrent neural network (RNN), allowing wide coverage of chemical space. We illustrate the RL training procedure of iMiner that uses on-the-fly AutoDock Vina^34^ in a predefined binding pocket of the 3D structure of the protein to generate small molecule inhibitors, in which the AutoDock-Vina score is used to adjust the RNN so that the distribution of generated inhibitor molecules are shifted toward those that more strongly interact with the protein. The iMiner algorithm is further distinguished from other generative models with additional capabilities to drive not only *de novo* molecular design but also ligand based design (generating similar molecules to early optimized hits) and structure based design (growth from bound ligand fragments and/or enforcing ligand interaction with certain protein sites), a unique versatility within our reinforcement learning. Finally, iMiner also offers the option to provide a series of postfilters to down-select the generated molecules so that they obey Lipinski rules,^35^ increase the likelihood of synthetic accessibility (SA) and drug likeliness, avoid nonspecific binders (Pan-assay interference compounds or PAINS)^36^ or certain disfavored substructure elements, and find consensus molecules with alternative docking software and scoring functions.

To validate the effectiveness of the iMiner approach, we designed inhibitors for the popular SARS-COV-2 Main protease (Mpro) as an example (see Supporting Information). We chose this system because of the ready availability of experimental 3D structures^37^ and the fact that multiple effective ligands have already been proposed to inhibit Mpro,^38^ which provides ample information for different structure based and ligand based designing strategies.^39^ We illustrate the four features of the iMiner approach and evaluate our model’s capability to generate new inhibitors and perform structural modifications while keeping the pharmacophore intact, growing ligands from a fragment starting point, or retaining key interactions with a specified protein residue. Although illustrated on the SARS-COV-2 Mpro target, we would like to emphasize that the iMiner workflow can be readily adapted to generate small molecules for other protein targets, since it only requires a 3D structure of the target protein with a predefined binding site. Thus, we believe our ML algorithm will be of great interest to the drug design community to rapidly screen novel regions of chemical space in real-time for other antivirals or small molecule therapeutics in the future.

## The iMiner Algorithm and Workflow

**Figure 1:**
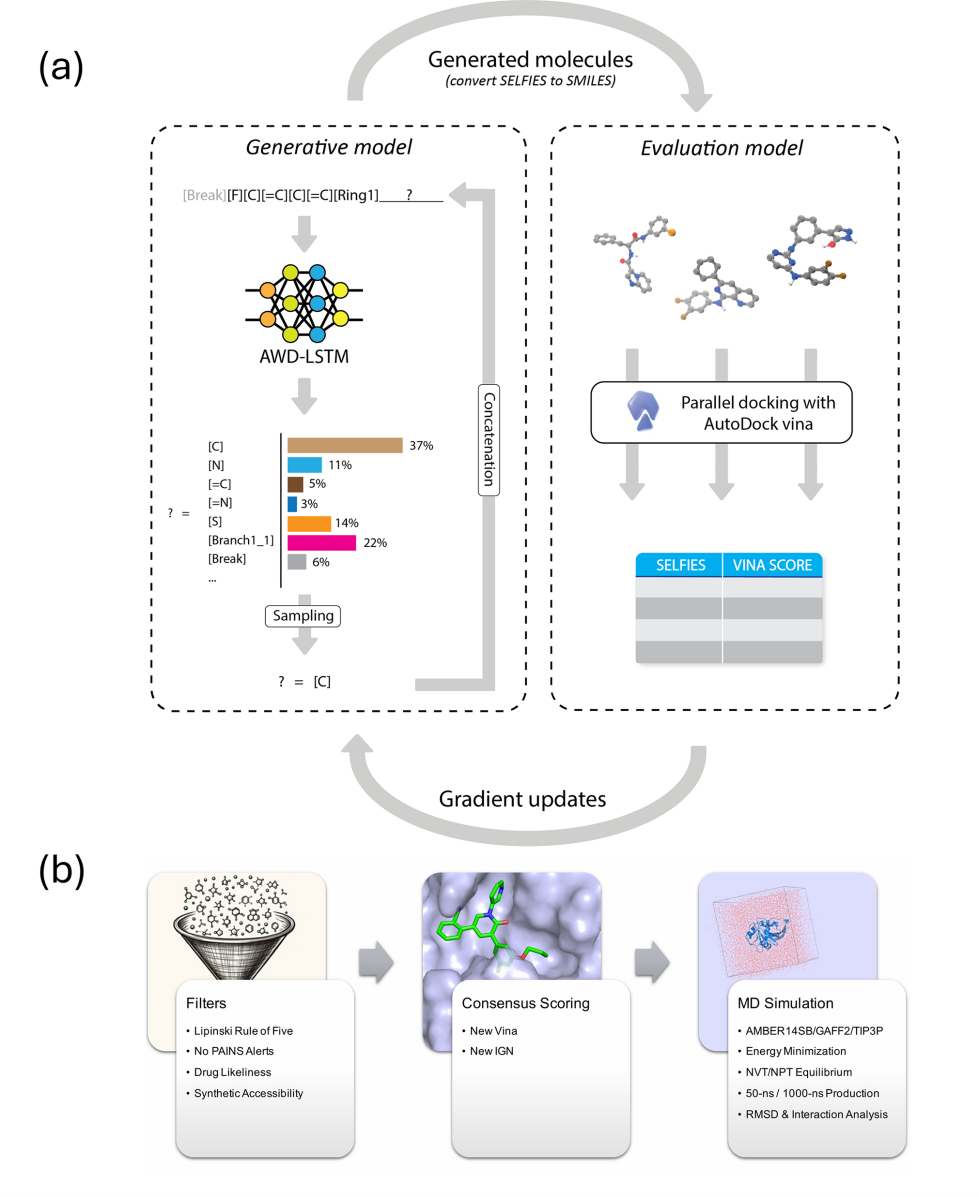
Illustration of the overall structure of the iMiner algorithm and workflow. (a) The generative model uses SELFIES representations for molecules and a recurrent neural network to “mine” for new molecules that are presented to the evaluative model for 3D docking using AutoDock Vina. Vina scores and other loss/reward functions are used to drive gradient updates of the neural network. (b) The iMiner workflow provides automated postselection filters, consensus scoring, and MD simulations for overall ranking.

Figure 1 provides an illustration of the overall structure of the iMiner algorithm, which highlights the two major machine learning components, the generative and evaluative models, and their interplay for generating new inhibitor molecules. In addition, we embed the RL-physics model into a composite workflow for further analysis and down-selection, also displayed in Figure 1. Here we describe the iMiner algorithmic components and workflow in more detail.

### The Generative Module

Conceptually, generating molecules using a string representation is similar to the way text is generated in a natural language processing task. Our method starts with a specific [Break] token, and for each molecule, we utilized a RNN that takes in the last token in the string, together with the hidden state from the last step to predict a distribution of tokens following the current string. In this work a specific variant of the RNN, known as the AWD-LSTM, was used due to its exceptional performance in similar generative tasks (Figure 1a).^33^

#### SELFIES Representation of Inhibitor Molecules

An arbitrary molecule can be represented as a topological graph using two main approaches: adjacency matrix methods and string based methods. The former uses an *N* by *N* matrix to encode a molecule, where *N* is the number of atoms in the molecule and the values of the matrix are typically bond orders between atoms. An adjacency matrix is not ideal for generative tasks because the size of the molecule that can be generated should not be fixed, and the learning of chemical knowledge by a ML model through adjacency matrix can be difficult. Instead, string based methods are more suited for molecular generation tasks, and SMILES strings have been the standard for molecular representation due to their conciseness and readability. However, SMILES strings have relatively complex syntax and require matching of open and closed brackets for branching, and ring modeling/modification is not trivial. Therefore, generating novel, chemically correct compounds through the use of SMILES strings can be challenging.

The SELFIES molecular representation^32^ is specifically designed to ensure that all generated strings correspond to valid molecules. By utilizing [Branch] and [Ring] tokens with predefined branch lengths and ring sizes, as well as generating symbols using derivation rules, the SELFIES representation guarantees that valence bond constraints are met and any combinations of tokens from its vocabulary correspond to a valid molecule. Therefore, we have used SELFIES in our generative model to encode molecules since it does not need to learn chemical syntax rules and can allocate more of its learning capacity toward generating valid molecules with properties of interest.

#### Pretraining the Inhibitor Molecule Generation

The network was pretrained using supervised-learning (SL) of all molecules from the ChEMBL database to learn the conditional probability distributions of tokens that correspond to drug-like molecules. When our trained generative model is used for generating new molecules, a new token is sampled according to the predicted probabilities, and this new token is concatenated to the input string to sample the next token until the [Break] token is sampled, in which case a complete molecule has been generated.

We then validated our pretrained distributions using 13 drug-likeliness properties between our generated molecules and randomly sampled molecules from the ChEMBL database that we used for training. The molecular properties considered are well-recognized chemical features related to the drug-likeliness of a molecule which can be obtained through 2D topological connectivity of the molecule: fraction of sp^3^ hybridized carbons, number of heavy atoms, fraction of non-carbon atoms in all heavy atoms, number of hydrogen bond donors and acceptors, number of rotatable bonds, number of aliphatic and aromatic rings, molecular weight, approximate log partition coefficient between octanol and water (alogP),^40^ polarizable surface area (PSA), number of structural alerts,^41^ and size of the largest ring in the molecule. Despite the fact that during pretraining only token distributions were used as training targets, all distributions collected from our generated molecules closely follow the distributions from the ChEMBL database (Figure S1). This result suggests our pretrained model has learned key concepts of “drug-likeness” and provides a good starting point for the RL procedure.

### The Evaluation Module

After our generative model was pretrained, we employed an RL workflow to bias the distribution of generated molecules toward specific properties of interest. RL training allows the model to interact with an environment by performing actions according to a policy model and uses the feedback from the environment to provide training signals to improve the model. In this work, the pretrained generative model is taken as the policy, and in each iteration, 2000 molecules were generated and sent to the evaluation module (Figure 1a).

#### Physics-Based Docking

The central component of our evaluation model is docking with AutoDock Vina (Vina) in parallel with the RL. Within our evaluation model, the Vina score calculator takes a SMILES string representing the ligand and the 3D structure of the protein target, together with a predefined docking region, as input. AutoDock Vina then explores variations of the dihedral angle degrees of freedom and identifies the optimal conformation of the input inhibitor for placement in the designated protein binding site. Finally, AutoDock returns the Vina score as an approximation of the binding energy between the ligand and the protein. Multiple instances of the Vina score calculator tasks were established through GPU parallelization to allow high-throughput evaluation of the generated molecules.^42^ Vina scores were then cycled back to the generative model to improve molecule generation through proximal policy optimization (PPO),^43^ as will be discussed in next section. We emphasize that by using a physics-based docking model which utilizes the full 3D structure of our target protein and generated molecules as the critic, the training of the policy model is less likely to be contaminated due to exploiting failure modes of a neural-network based critic, an issue called wireheading.^44^ Instead, we benefit from a more reliable training signal and reduce the false positive and false negative rates of the generated molecules.

Vina scores alone are not sufficient to reliably train a molecule generator, as shown in the Supporting Information (Figure S2) because it will not always satisfy requirements for drug-likeness. To ensure that our generated molecules still bear drug-like properties, we incorporated an additional metric into the reward, *S*_DL_, which is a weighted average of the log likelihood for the 13 different drug-like properties used in pretraining assessment. Our custom drug-likeliness score is an extension of the widely used quantitative estimate of drug-likelihood (QED) value^45^ tailored to our deep learning generative model. Formally, the drug-likeliness score *S*_DL_ is defined as
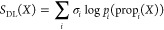
1where prop_*i*_(*X*) calculates the *i*th property of a molecule *X* and *p*_*i*_ is defined by the probability distribution of property *i* by all molecules in the ChEMBL database. The parameter σ_*i*_ is defined as
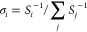
2where *S*_*i*_ is the entropy of the distribution of property *i*,
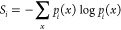
3such that a narrower distribution from the ChEMBL database contributes more to the drug likeliness score and defines the weights for each property as proportional to the inverse of the entropy. Introducing this additional reward ensures our model also accounts for similarity of generated molecules to the drug-likeliness present in the ChEMBL database and ensures that our generated molecules are more likely to be optimal leads for further drug design endeavors. Additionally, since the ChEMBL database also contains molecules with undesirable molecular scaffolds, we allow users to define specific undesirable chemical patterns using SMARTS strings^46^ and manually set the drug-likeliness score to 0 to discourage their appearances in future iterations. A set of suggested SMARTS patterns have been enabled by default, and further details are given in Supporting Information and Table S1.

#### Reinforcement Learning with Different Reward Types

Our pretrained policy model defines a probability distribution for an arbitrary sequence of tokens from the SELFIES vocabulary, since the generation of the sequence is a Markovian decision process (MDP), and can be written as
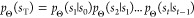
4where *s*_0_ corresponds to a starting state with [Break] as the only token in the string, *s*_*t*_ corresponds to an intermediate state with a finite length string of SELFIES tokens not ended with the [Break] token, and *s*_T_ corresponds to the terminal state, with the last token being [Break] or the length of the string exceeding a predefined threshold. *p*(*s*_*t*_|*s*_*t*–1_) is the transition probability at time step *t*, which is the probability distribution of the next token from the generative RNN with the network parameters Θ. For each terminal state not exceeding the length limit, a corresponding molecule can be decoded, and the Vina score *S*_vina_ and drug-likeliness score *S*_DL_, can be calculated and further optimized. The total reward for a terminal state with a decoded molecule *X* is then defined as
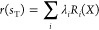
5where *i* denotes the reward function types and λ parameters control the balance between the different scores. For the unconditional *de novo* generation case, where only the drug-likeliness score needs to be maximized and Vina score needs to be minimized, the reward becomes
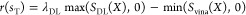
6Negative *S*_DL_ is upward clipped to 0 and positive *S*_vina_ is downward clipped to 0 to ensure the reward is non-negative. The expected reward under the MDP is then
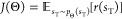
7

To encourage the model to generate molecules based on a certain scaffold, we introduced a fragment similarity score, defined by the maximum Dice similarity of the Morgan fingerprints between fragments of the query molecule decomposed using BRICS^47^ and the target molecule or structural components, as well as a pharmacophore similarity score with the Pharm2D^48^ module in RDkit.^49^ In addition to molecular similarity, we also implemented a positional penalty of the docking score to impose a 3D structural similarity if needed. The algorithm identifies the most similar fragment component in a sampled molecule, calculates the shape alignment of the component to the target fragment, and clips the docking score to 0 if the alignment difference exceeds a given threshold.

A further augmented feature of iMiner is the generation of molecules that interact with specific protein residues in the pocket. We developed an interaction score as the weighted total of interactions of specific types between the predicted binding pose of a sampled molecule and the specific residues using Protein–Ligand Interaction Profiler (PLIP),^50^ where the weights are defined by users to prioritize certain interaction types and/or protein residues as desired. We also differentiated hydrogen bonding with angle <135° and the hydrogen-donor bond length >2.8 Å as weak interactions and down-scaled the corresponding interaction scores by 0.5 to encourage the generated molecules to form stronger bonds.

While we present results below using the different reward scores separately for clarity, all the above customized scores can be used jointly along with the drug-likeliness score and Vina score to meet different design criteria. Further details of the RL training procedure and hyperparameters λ are given in the Methods section and Supporting Information and Table S2.

### The iMiner Workflow

The iMiner algorithm is embedded in a composite workflow that automates the postanalysis of generated molecules to help with overall ranking (Figure 1b) After completion of molecule generation and optimization, it is desirable to filter out PAINS molecules as well as molecules with Lipinski rule violations^51^ and to select for molecules with good synthetic accessibility (SA) scores according to the SwissADME software.^52^ We therefore developed and applied a set of filters requiring no PAINS, no Lipinski rule violations, and SA scores using the RDKit package,^49^ as well as a drug-likeliness score filter of >2.8 to exclude any structure that is not sufficiently drug-like. For the task to generate molecules with desired protein–ligand interactions, we applied an additional filter to extract molecules with confirmed interactions with the specified protein residue(s). While we use these specific values for the results described below, users have the option to change these values if desired.

Checking for consensus with alternate scoring functions is often considered good practice as any individual scoring function may have limited accuracy or be parametrized for different test cases.^53^ Thus, the workflow collects all molecules from RL training iterations with a user-defined Vina score cutoff and evaluates them with two alternative scoring functions. For cross-validation, we utilized a new AutoDock Vina score (newVina) and new InteractionGraphNet (newIGN)^54^ that were recently retrained using LP-PDBBind,^55^ a cleaned version of PDBBind data that implement control on the data similarity between the train, validation, and test data set. It was shown that the predicted binding affinities using retrained Vina and IGN scoring functions have much better accuracy and generalizability than those from the old versions, when evaluated using the cocrystal structures.^55^ The next step was to rescore the above resulting molecular poses with the newVina and newIGN scores and extract molecules that are in a certain consensus top range.

The final stage of the iMiner workflow is to run a molecular dynamics (MD) simulation for the protein–ligand complex. It has been widely recognized that molecular docking has some difficulties in terms of distinguishing nonbinders from binders as a consequence of lacking ensemble information and inability to capture induced-fit effects. Physics-based MD simulation has demonstrated its ability to improve the power of classical docking functions by effectively incorporating flexibility of both the receptor and the ligand while also including molecular aqueous solvent. Specifically, Guterres et al. have shown that 50 ns MD can serve as a postfilter for docking outcomes to distinguish decoys from binders if the ligand’s RMSD during the trajectory is larger than a threshold, which means that the binding is less stable.^56^ Therefore, we incorporate MD simulations into iMiner as a tool to validate the protein–ligand binding.

The automated MD procedure is described as follows: (1) the protein–ligand pose with the best Vina score is used as the initial structure and the simulation system is prepared by adding water solvent molecules and when required neutralizing counterions (Na^+^ or Cl^–^); (2) AMBER14SB/GAFF2/TIP3P are adopted to parametrize the protein, ligand, and water, respectively; (3) the system is first energy minimized followed by a 1 ns equilibration simulation in the NVT ensemble under 1 bar, 298.15 K, followed by a 1 ns simulation under the NPT ensemble; (4) a 50 ns production run is performed in the NPT ensemble and a total of 250 structures are collected for analysis in step 5; (5) for each structure the ligand’s RMSD is calculated with respect to the starting pose, and the probability of interactions between the ligand and protein are also analyzed with PLIP;^50^ (6) for a stable protein–ligand complex from step 5, if the user specifies, a longer production run in the NPT ensemble can be performed and the simulation time is also determined by the user. Herein, we use 1 μs for our SARS-CoV-2 main protease target in which the active site is not clearly defined solvent-exposed (not cryptic).^57^ For those targets that contain cryptic pockets or the structure is flexible, longer time may be needed.

## Results

### Unconditional *De Novo* Generation

We use SARS-CoV-2 Mpro as an example to demonstrate iMiner’s ability to generate a diverse set of potential small-molecule drug-like candidates without any information beyond the active site. To achieve this goal, two objectives are optimized during the RL training: (1) AutoDock Vina docking score to ensure the proposed molecules have good binding efficacy and (2) drug-likeliness to constrain our chemical search space only to molecules that are considered as drug-like.

**Figure 2:**
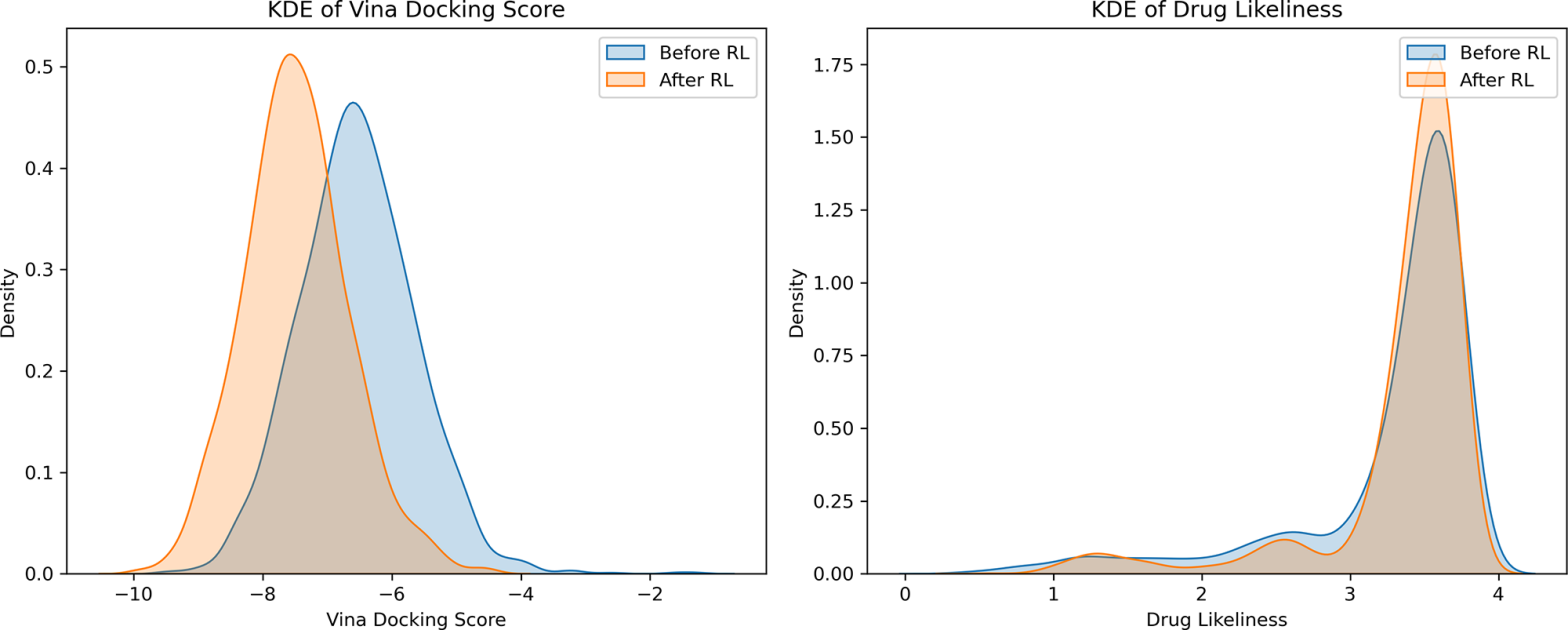
Kernel density estimate plots of Vina docking score and drug likeliness for *de novo* generated molecules before and after RL training. For this comparison, 1000 molecules were generated using the models before and after RL training to create the plot.

When the training was completed, we looked at the shift in the distributions of the optimized objectives and the molecular properties of generated compounds. In Figure 2, we compare the distribution of Vina score and drug-likeliness score before and after RL training. The clear shift toward more negative Vina scores while maintaining a similar drug-likeliness distribution shows that iMiner can bias the generative model toward more potent and drug-like binders.

Furthermore, to inspect the changes in molecular properties before and after RL optimization, in Table 1, we evaluated the GuacaMol benchmarks,^58^ which probe 5 different aspects of the distribution of generated molecules with respect to the training data set. Model “validity” reports the proportion of syntactically correct molecules. Because we generated molecules via SELFIES representations, we achieved close to 100% validity for all generated molecules both in the pretrained step and after the RL optimization. Invalid molecules were either empty strings or molecules for which the SELFIES package failed to convert into a SMILES string and therefore were discarded before the next workflow steps. Model “uniqueness” reports how many generated molecules are duplicates versus those which are genuinely distinct. Our pretrained and RL models illustrate high uniqueness, indicating the model is able to generate a wide variety of nonredundant molecules. Model “novelty” is defined as the proportion of generated molecules that do not exist in the training data set. The high novelty of both our pretrained and RL model indicates that it is not memorizing molecules from the training data set but is indeed generating molecules that it has not seen before. By these 3 metrics, the pretrained and RL molecules satisfy the criteria of an unconditional *de novo* generation of unique molecules.

**Table 1:** GuacaMol Benchmarks for the Pretrained Generative Model and after RL Training^a^

benchmark	pretrained model	after RL
validity	0.998	0.998
uniqueness	0.999	0.994
novelty	0.867	0.952
KL divergence	0.985	0.855
Frechet ChemNet Distance	0.870	0.301

a The model benchmarks include valid chemical molecules, uniqueness and novelty with respect to the training set, and distribution similarity evaluated using KL divergence and Frechet ChemNet distance.

But the RL learning should also generate unique molecules that are specific to the 3D interaction space of the binding pocket. The Kullback–Leibler (KL) divergence measures differences in probability distributions of various physicochemical descriptors for the training set and the pretrained and RL model generated molecules. As defined by GuacaMol, a high KL divergence benchmark suggests that the generated molecules have similar physicochemical properties to those of the training data set. This is true for the pretrained models by design, but what is clear after the RL optimization is that the influence of the 3D shape requirements of the protein pocket and emphasis on drug-likeness results in significant deviation from the original training set. This is also reflected in the Frechet ChemNet Distance (FCD), which utilizes a hidden representation of molecules in a previously trained NN to predict biological activities and thus captures both chemical and biological similarities simultaneously for two sets of molecules.^59^ Molecules generated by our pretrained model have high FCD values, indicating that our molecules have similar biological activities as molecules from the ChEMBL training data set. However, the strong deviation after RL training shows that the generated molecules reside in a different chemical space. In Table S3, we show that the model after RL training can generate molecules that are more similar to those of known SARS-Cov-2 Mpro binders.

After the initial filtering with a top 10% Vina docking score cutoff, 12,600 molecules were selected for consensus rescoring and chemical property comparisons with other deep-learning-based methods were shown in Table S4. Then, 125 molecules were filtered from the consensus scoring for the final analysis to assess their distribution compared to a set of the known experimental binders borrowed from LP-PDBBind.^55^ Specifically, we looked at the newVina and newIGN score distributions as well as the bioactivity scores used in similar studies.^60,61^

**Figure 3:**
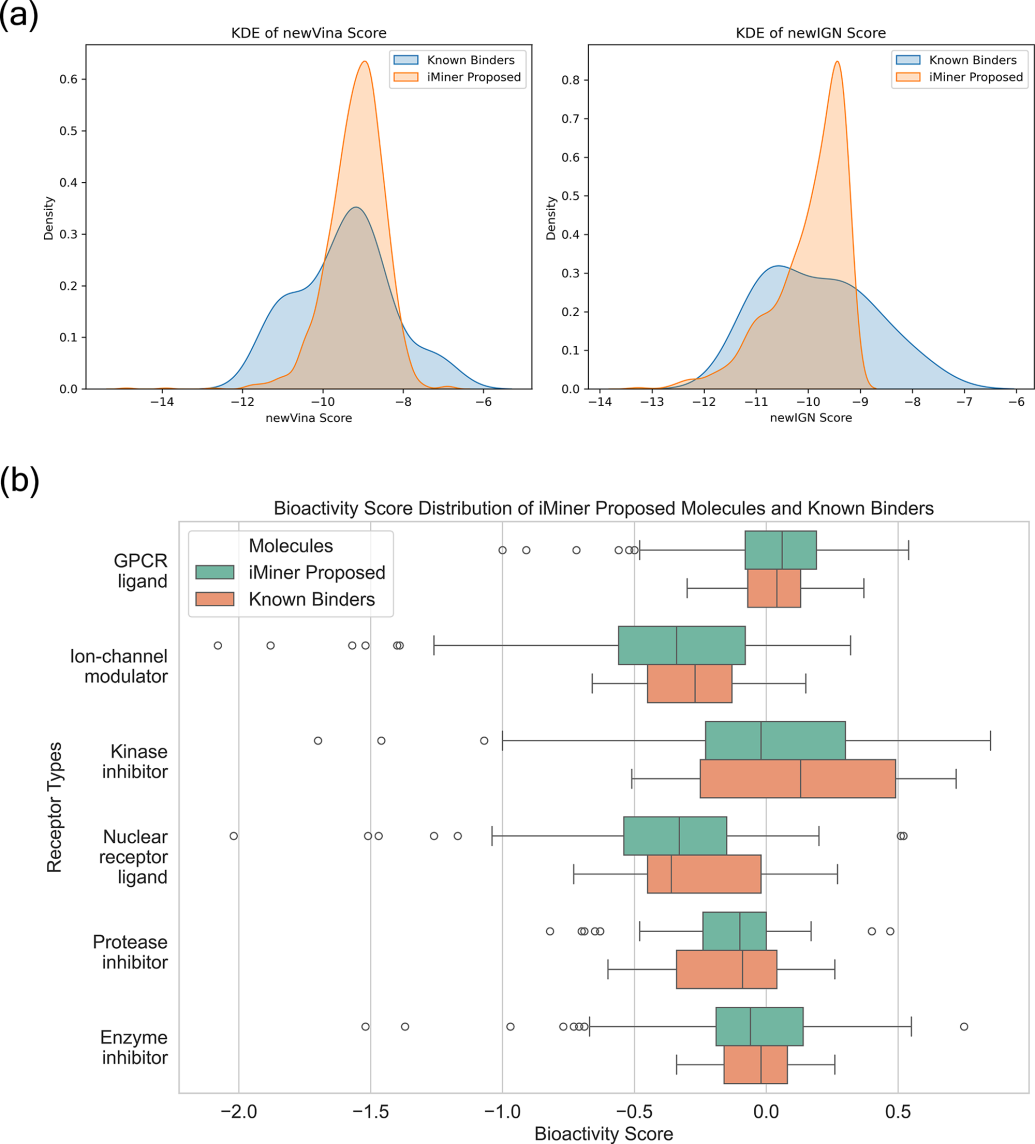
Drug-related property analysis between iMiner proposed molecules and experimentally confirmed binders. (a) Kernel density plots of the newVina and newIGN score distributions across two groups of molecules. (b) Distribution comparison of six different categories of bioactivity scores for the predicted target interaction.

In Figure 3a, the distribution of the surviving molecules falls in a similar and, in the case of newIGN, even better range compared to the known experimental binders, indicating that our stringent down-selection with consensus scoring could successfully lead us to molecules that have the potential to form strong interactions with our target protein. In Figure 3b, we examine the various bioactivity scores to show that our molecules are similar to the known binders from a pharmacological perspective. Bioactivity scores are a quantitative estimate of a compound’s interaction with different targets. A score of less than −0.5 usually means the compound is inactive; a score in the range of −0.5 to 0 corresponds to the compound being moderately active; and a score of larger than 0 indicates the compound is biologically active.^60^ Here, this similarity in the score distribution across all targets indicates that the proposed molecules and the experimental binders share close pharmacological properties. Specifically, 29 proposed molecules have a bioactivity score larger than 0 in the category of protease inhibitor, suggesting a high potential for proposed compounds to be effective.

### Generation of Structural Analogs

Structural analogs are designed to have a similar structure to a reference molecule which is (often) a known binder but made to differ in certain functional groups to optimize the structure–activity relationship (SAR). Developing structural analogs is valuable in drug discovery because they can display a spectrum of biological activities distinct from the parent molecule, but it remains a combinatorial search challenge such that automated computational approaches can be of great value. We therefore have adapted iMiner’s reinforcement learning coupled with the docking algorithm to also include a structural similarity score to efficiently explore the variations in chemical space around a given target compound. We have considered two types of structural similarity: fingerprints that encode the topology of a molecule and pharmacophore models that capture the arrangement of chemical features important for biological activity. In this work, we exemplify this approach by generating analogs of the SARS-CoV-2 Mpro inhibitors reported by Zhang et al.^38^

**Figure 4:**
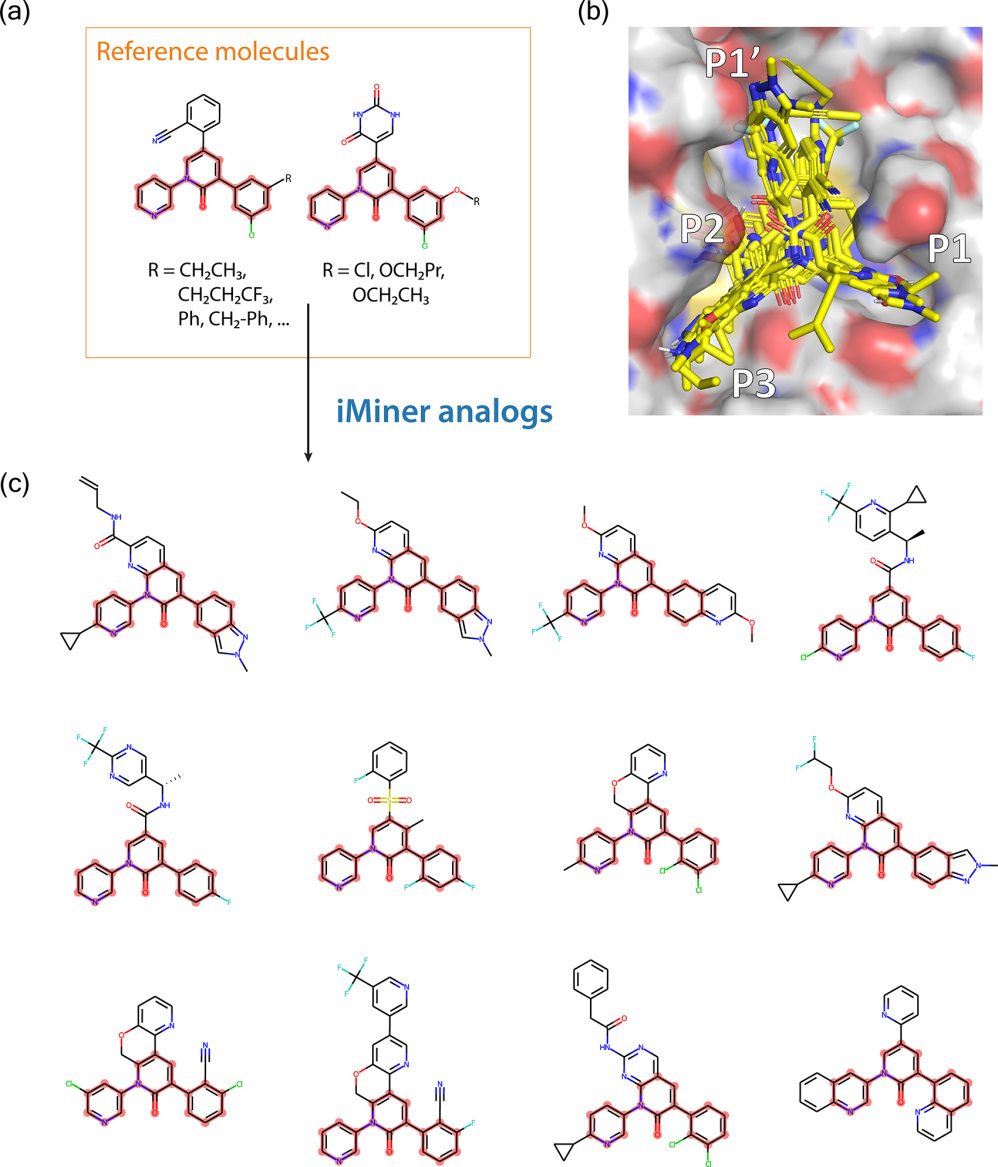
iMiner generated structural analogs from a set of reference molecules. (a) The perampanel-derived^38^ reference molecules used to guide iMiner generation. (b) Surface rendering of the iMiner generated analogs in the Mpro canonical binding pocket. (c) 2D visualization of the iMiner-generated analogs with the shared motif highlighted in pink. Also see Table S5.

Figure 4a displays the reference molecules which are comprised of a core cloverleaf motif around a central pyridinone ring, a pyridinyl group, and a meta-substituted phenyl ring directed toward His41, features considered to be well-optimized in ref (38). As seen in Figure 4b, the iMiner molecules generated to maintain similarity to the core motif have also varied the functional groups extending into the P3 region of the Mpro canonical binding pocket, as seen in Figure 4b. In addition, Table S5 shows that both newVina and newIGN scores of the docked poses of the generated molecules determined by Autodock Vina aggregate in the more negative energy (higher affinity) range compared to those of the reference perampanel derivatives since the iMiner model also optimizes binding affinities while exploring a local chemical space around the reference molecules.

### Structure-Based Fragment Growth

Fragment-Based Drug Discovery (FBDD) has emerged as an attractive strategy in modern medicinal chemistry and drug development due to its advantages in lower experimental cost and diversity of paths to novel compounds.^62^ Fragments, for instance, from a screening campaign or as an interesting substructure of a validated binder, can serve as foundational building blocks which can be evolved and optimized into potent drug candidates. While fragments provide only starting points due to their modest affinity, deciding how to grow the molecule or even connect multiple fragments while enhancing activity can be an intricate and subtle task. While FBDD can be readily addressed by iMiner using structural similarity scores as demonstrated above, it is now more challenging because we are also enforcing the structural binding mode of the starting fragment. One intuitive approach would be to perform docking with constraints. However, few publicly available docking programs offer such a function without extensive reparametrization. Instead, iMiner employs a “top-down” approach that in addition to rewarding molecules with high similarity, also penalizes the docking scores of molecules whose binding poses do not match with the starting fragment, thereby reducing sampling of molecules with incorrect orientation or connectivity during the RL phase.

**Figure 5:**
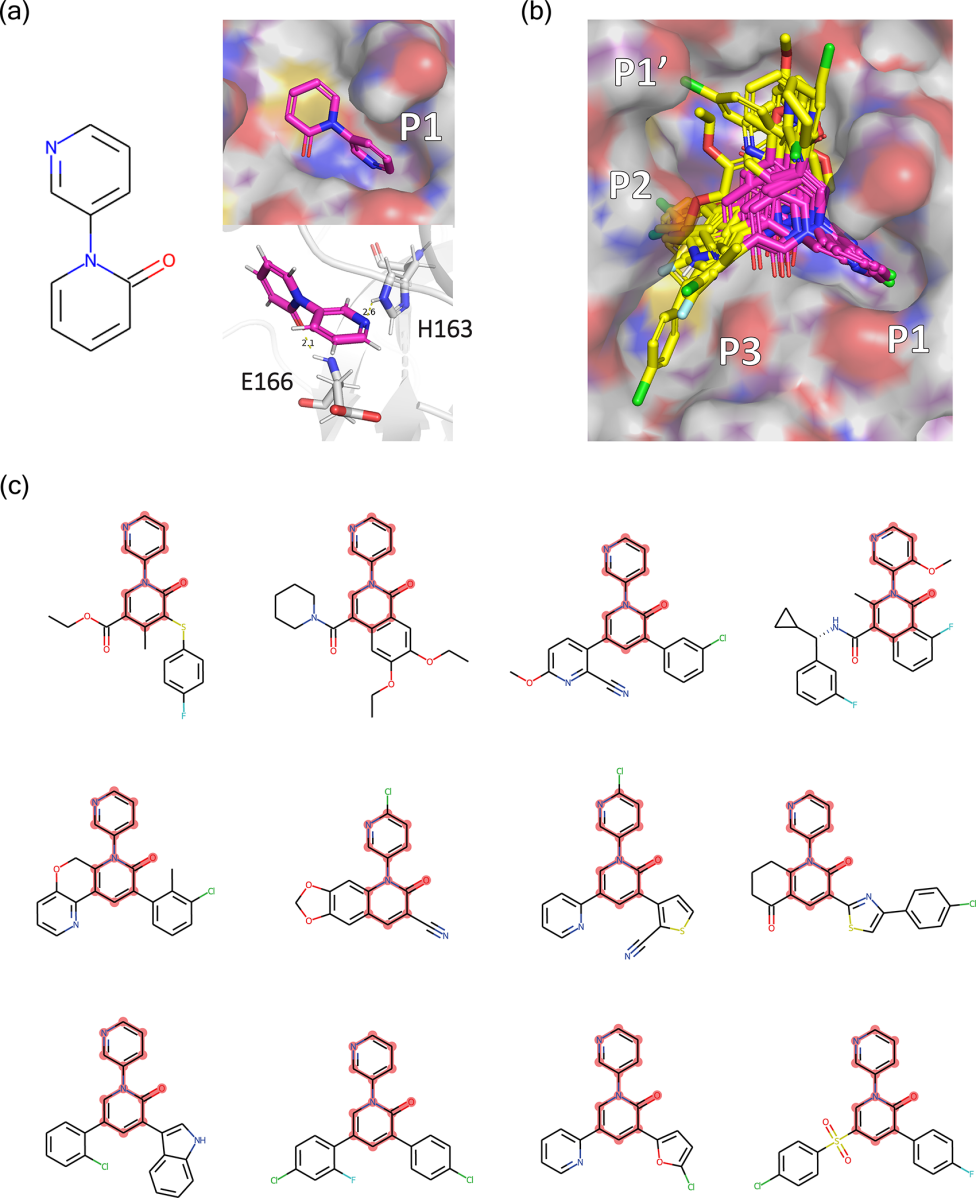
Structural based fragment growth using iMiner. (a) 2D and 3D visualization of the core fragment sitting in the pocket and interacting with His163 and Glu166. (b) Surface rendering of the filtered set of iMiner generated molecules overlaid in the Mpro canonical binding pocket denoted as P1, P1′, P2, and P3. The substructure fragments are shown in magenta. (c) Twelve representative filtered molecules with the fragment highlighted. Also see Table S6.

As an example, we use iMiner to propose molecules containing a pyridinone ring connected to a pyridinyl group that resides in the Mpro P1 pocket (Figure 5a), extracted from the molecules developed by Zhang et al. for Mpro, by performing RL training with a pose adjusted Vina score and fragment similarity score starting from a fine-tuned model (see Methods section). A set of 12 representative molecules in Figure 5b,c with the matching substructure aligned to the starting fragment were harvested from the validation and postfiltering process. As seen in the figure many of the proposed molecules extended from the fragment adopt a cloverleaf-like motif branching into the P1, P1′, and P2 subpockets as observed in Zhang et al. The preservation of hydrogen bonding interactions to His163 and Glu166 of these predicted poses, as adopted by the original fragment, was also preserved in the iMiner molecules. Together the results verify that the RL-physics model not only learned the chemical and structural information on the fragment but was able to grow a larger molecule that improves the pocket complementarity.

### Designing Molecules to Interact with Specific Protein Sites

An alternative scenario of significant interest involves designing molecules to engage with specific protein residues within the pocket. These residues may possess vital catalytic activities or exhibit low mutation rates; thus, the inhibitors designed specific to these residues are less likely to develop resistance to viral variants. We investigated the use of iMiner to generate molecules with hydrogen bonding interactions to His163 in the SARS-CoV-2 active site. His163 is a conserved residue in different SARS-CoV-2 strains and multiple inhibitors, such as the FDA-approved remdesivir^63^ and ensitrelvir,^64^ show consensus in forming a hydrogen bond with His163. Starting from the ChEMBL pretrained model and assuming no other knowledge about the structural and chemical features of a potential Mpro binder, we trained a model using an interaction score with respect to key residues such as His163 and Glu166, Vina docking score, and drug-likeliness score in the reward function.

**Figure 6:**
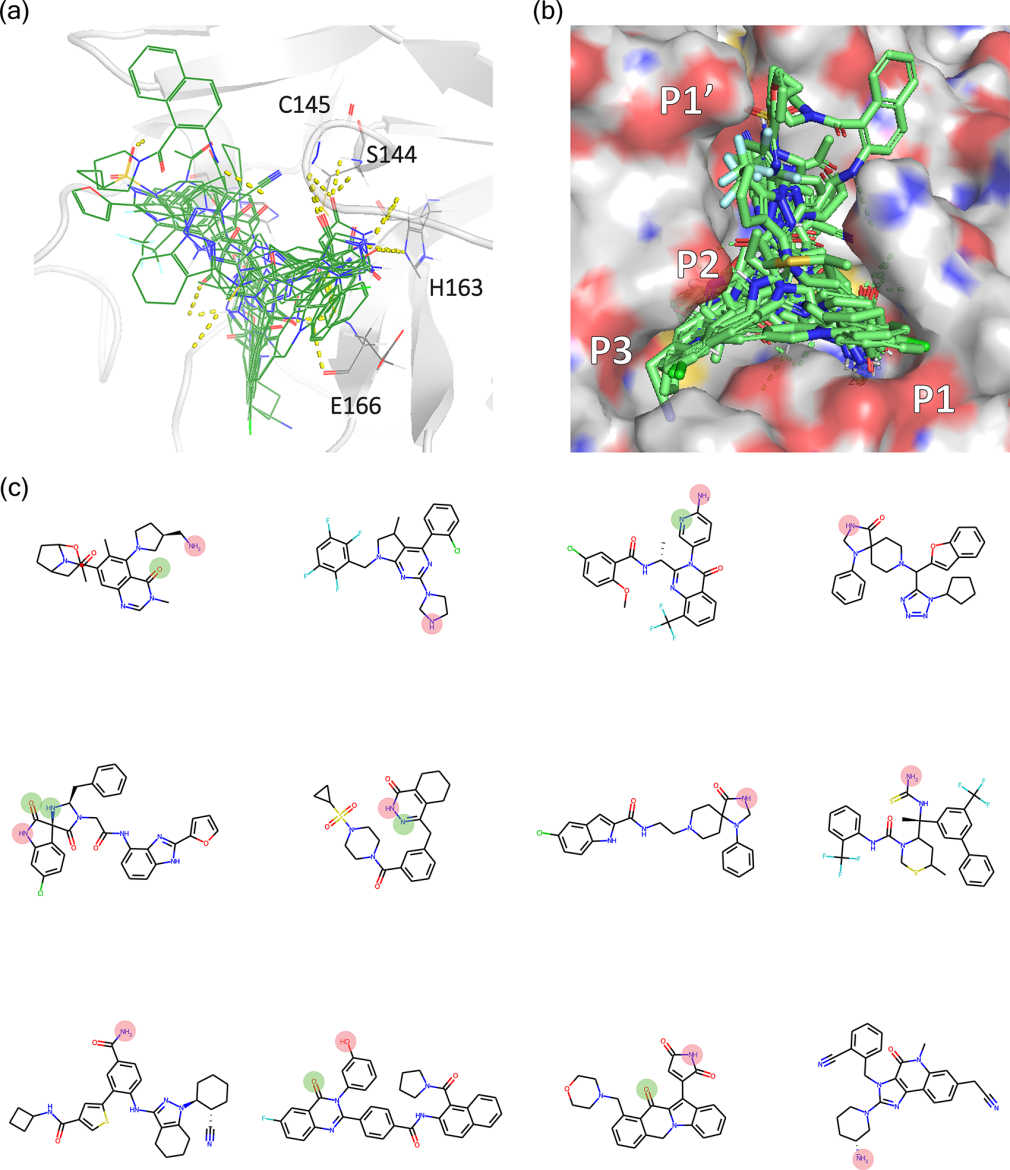
Molecules from iMiner interaction based design to target Mpro His163/Glu166. (a) Binding modes of the generated molecules show a rich polar interaction profile against specified residues. (b) Surface rendering of the generated molecules in the Mpro canonical binding pocket. (c) Twelve representative iMiner molecules with highlighted hydrogen bond donor/acceptor groups that form interaction with His163 (pink) and Glu166 (green). Also see Table S7.

In Figure 6, the overlay of postfiltered iMiner-generated molecules in their binding conformations determined through AutoDock Vina confirms their participation in hydrogen bonding interactions with His163 or Glu166 or both as expected. In Figure 6a, this set of 12 representative molecules has shown a diverse set of scaffolds while retaining the key interactions with the specified residues. Figure 6b further shows that the molecules could fill the P1, P2, P3, and P1′ subpockets, satisfying the pocket complementarity. In addition, several other key hydrogen bonding interactions with the backbone atoms of Cys145 and Ser144 were recovered from the docked poses of the proposed molecules, although these interactions were not explicitly enforced by the reward function.

### Binding Stability Analysis

**Figure 7:**
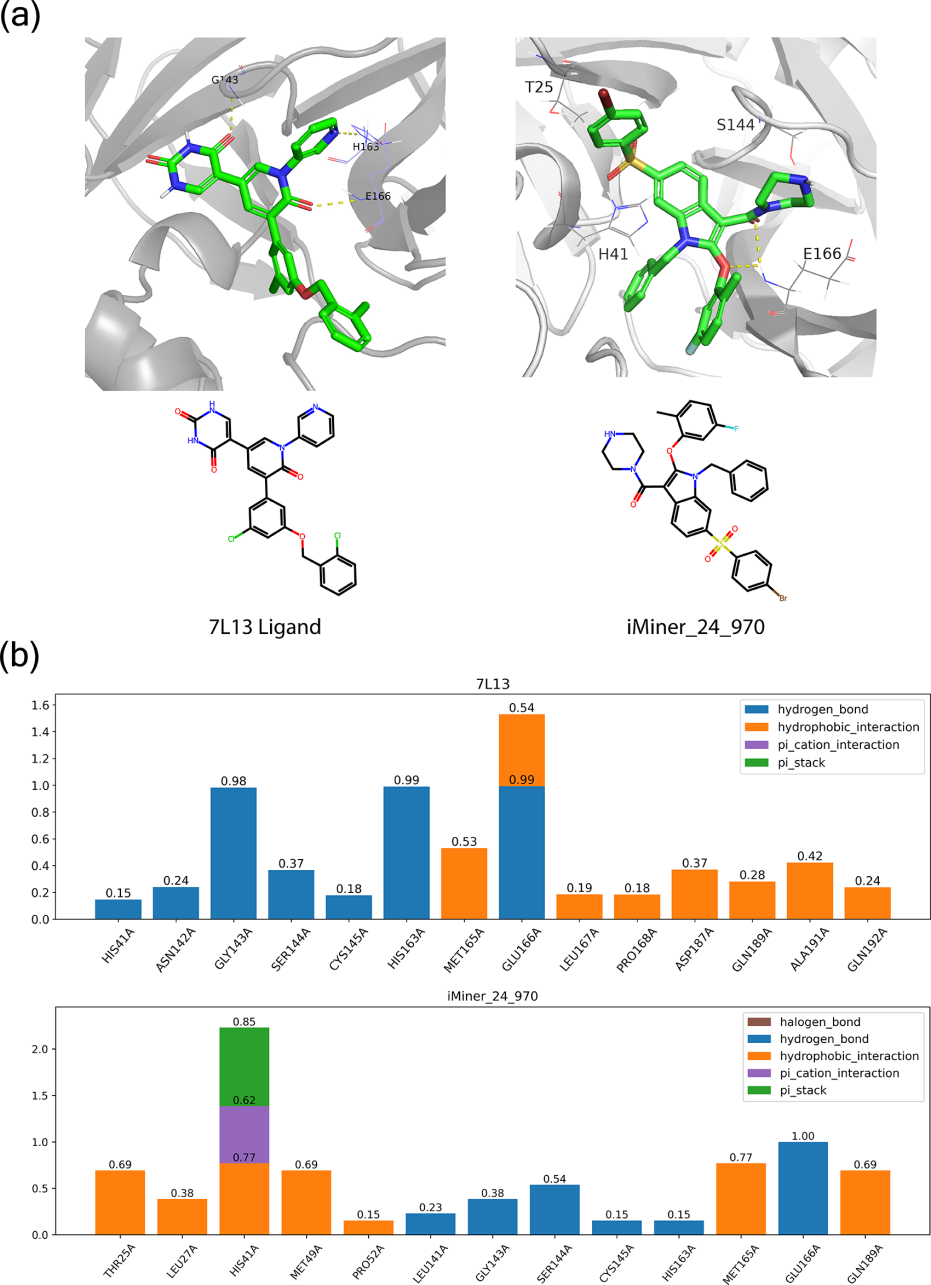
Binding stability analysis between an iMiner-proposed molecule and a known ligand. (a) Representative 2D and 3D visualization of the crystal ligand of Mpro (PDB Code: 7L13) from Zhang et al.^38^ and iMiner_24_970 from the unconditional *de novo* design. (b) Residue-level protein–ligand interaction frequency comparison performed between both molecules.

As the final step of the workflow following consensus scoring, we perform subsequent long MD simulations to measure the stability of the surviving molecules. The effectiveness of using MD to validate binding through experiment has also been demonstrated before.^65^ To illustrate this stability test, we analyzed the *de novo* generation design set, which consisted of 12 down-selected molecules from a more stringent newVina and newIGN rescoring threshold of the top 1.5% in each category. For each molecule, we start with 50 ns MD simulations, from which we found that 7 molecules had robust binding stability as defined by an average RMSD smaller than 4 Å and preserving more than 1 hydrogen bond between the ligand and protein residues. To validate the MD results for these 7 molecules more rigorously, the simulation was then prolonged to 1 μs based on previous studies of Mpro.^57^ In Figure 7, we show the best out of 3 *de novo* generated molecules that retained good binding within the active site. Further ligand trajectory-based analyses of other molecules proposed by all four protocols are shown in Figures S3 and S4. For comparison, we also performed 1 μs simulation for two known binders (PDB code: 7L11 and 7L13) reported by Zhang et al.^38^

As is evident in Figure 7 and Figures S3 and S4, the iMiner-generated molecules form very stable interactions such as hydrogen bonds, hydrophobic interactions, and pi-stacking, just as is the case for the known binders 7L11 and 7L13.^38^ In Figure 7 we find that both 7L13 and iMiner_24_970 form stable hydrogen bonds with Glu166, which is desirable as it is a low mutation site residue in Mpro such that these molecules may remain viable against future variants of SARS-Cov-2.^66,67^ The iMiner molecule also forms a long-lived pi–pi stacking interaction with His41, while the hydrogen bonding interaction with His41 is only weakly populated in 7L13. Similar conclusions are drawn when comparing the known binders against other iMiner proposed molecules. Thus, at the end of the iMiner workflow, the 3 generated molecules are certainly worth pursuing for synthesis and validation against biochemical and cellular assays.

## Conclusions

In this work, we have shown that by combining real-time docking of 3D structures with state-of-the-art reinforcement learning algorithms, we can efficiently navigate through uncharted regions of chemical space while maintaining good metrics for synthetic feasibility and drug-likeness. The flexibility of the iMiner physics-ML model also allows for the creation of molecules that enforce interactions with target active site residues as well as growing molecules from fragments with options for satisfying chemical or structural restraints. As illustrated using the exemplar target, the Mpro catalytic site, the generated inhibitor molecules proposed by iMiner are optimized with respect to shape and intermolecular interactions to the target protein, but are also diverse enough when compared to other predicted Mpro inhibitor data sets, i.e., experimentally confirmed molecules extracted from LP-PDBBind,^55^ especially the trefoil inhibitors optimized by the Jorgensen group.^38^ Finally, every aspect of this work is generalizable to other protein targets and beyond the active site, for example, allosteric sites.

Overall, we believe the iMiner RL-physics algorithm and workflow tool will be of great benefit to the computational and medicinal chemistry fields at large and potentially aid traditional drug-design workflows as well. Although we have focused our current work on targeting Mpro of SARS-CoV-2, extension of this work to other protein targets relevant to other global diseases would be relatively trivial. For example, bacterial resistance to antibiotics is of preeminent concern in the medical community,^68^ and our iMiner workflow approach could be used to target novel bacterial biomolecules, such as bacterial ribosomes, or target resistance conferring bacterial proteins such as β-lactamase.^68^ Another direction pertains to molecules that are experimentally validated through a traditional HTVS approach as good binders, in which the iMiner algorithm could be utilized as an optimization or refinement step for elaborating on these existing leads or scaffolds. The potential of the method in this direction will be explored in future work.

## Methods

### Neural Network Architecture

The generative model employed in this study is an ASGD Weight-Dropped LSTM (AWD-LSTM),^33^ which is a specific variant of the Long Short-Term Memory (LSTM) recurrent neural network with shared DropConnect for weight regularization, and was trained through a non-monotonically triggered average stochastic gradient descent (NT-ASGD) algorithm.^33,69^ The basic LSTM cell contains two internal states, the hidden state *h*_*t*_ and the cell state *c*_*t*_, and can be described through the following set of equations:
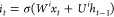
8
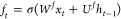
9
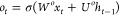
10
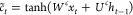
11
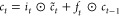
12
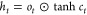
13where [*W*^*i*^, *W*^*f*^, *W*^*o*^, *W*^*c*^, *U*^*i*^, *U*^*f*^, *U*^*o*^, *U*^*c*^] are the trainable parameters of the model, *x*_*t*_ is the input to the cell at the current time step, *c̃*_*t*_ contains the information to be added to the cell state, and *i*_*t*_, *f*_*t*_, and *o*_*t*_ represent the update gate, forget gate, and output gate, respectively, which are numbers between (0, 1) that control how much information should be updated, discarded, or retrieved from the cell state. σ denotes the sigmoid function, and ⊙ represents element-wise multiplication. The DropConnect mechanism^70^ was applied to the hidden-to-hidden weight matrices [*U*^*i*^, *U*^*f*^, *U*^*o*^, *U*^*c*^] by randomly zeroing out a small portion of the parameters in these weight matrices to prevent overfitting and ensured that the same positions in the hidden vectors were treated consistently throughout the forward and backward pass in regards to whether or not to be dropped.

The inputs into the RNN cells were tokens embedded as vectors of length 400, and 3 LSTM cells were stacked sequentially, that had 1152, 1152, and 400 units each. The hidden state from the last time step of the last RNN cell was then connected to a linear decoder with output size of 56 and softmax activation, representing the probabilities of the 56 possible tokens from the vocabulary. The dropout values used in the model were embedding dropout = 0.002, LSTM weight dropout = 0.02, RNN hidden state dropout = 0.015, and output dropout = 0.01. The neural network was implemented using pyTorch^71^ and the fastai package.^72^

### Supervised Pretraining of the Network

The generative model was pretrained using molecules from ChEMBL24,^5^ and a total of 1,440,263 molecules were selected for training. All molecules were first converted to self-referencing embedded strings (SELFIES) using the selfies python package,^32^ and the tokens were extracted from the SELFIES with the fastai language model. We used categorical cross entropy loss:
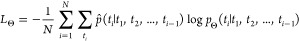
14where *N* represents the number of tokens in a molecule, *p̂*(*t*_*i*_|*t*_1_, *t*_2_, ..., *t*_*i*–1_) represents the actual probability of a specific token in the string at position *i* and with all previous defined tokens *t*_1_ through *t*_*i*–1_, and *p*_Θ_(*t*_*i*_|*t*_1_, *t*_2_, ..., *t*_*i*–1_) represents the probability predicted by the neural network with parameters Θ. The model was trained using Adam optimizer^73^ in batches of size 512, and we employed the “one cycle” learning rate policy^74^ with the maximum learning rate of 0.0005 to achieve superconvergence.^75^ During this pretraining stage we also used weight decay = 0.01 and the dropout multiplier of 0.2. The model was pretrained for 30 epochs.

For iMiner used to generate molecules similar to a scaffold or growing from a fragment, fine-tuning was conducted using molecules containing the target scaffold or fragment. These molecules can be curated from open source databases via a substructure search or from a set of molecules of any size defined by the users. While it is possible to directly perform RL training from the ChEMBL pretrained model with a fragment similarity reward term, the additional training ensures a consistent model performance in finding molecules with the desired substructures, as otherwise some specific fragments can be rare in the ChEMBL24 data set and the model cannot make sufficient sampling of the relevant structures in the initial few epochs of RL training. Details of the additional fine-tuning are provided in the Supporting Information.

### Reinforcement Learning Procedure

Our RL training target goal is to maximize *J*(Θ) from formula 7 by taking steps along ∂_Θ_*J*(Θ). The exact value for *J*(Θ) is intractable to evaluate but can be approximated through sampling the distribution of *s*_T_, which gives
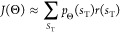
15and then
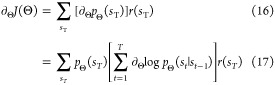
17Directly taking gradients according to eq 17 corresponds to the REINFORCE algorithm.^76^ In this work, we further utilized the PPO algorithm,^43^ which estimated the gradients through a clipped reward and with an extra entropy bonus term:
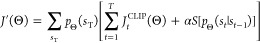
18where
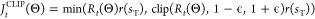
19with
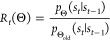
20denoting the ratio between the probability distribution in the current iteration and the probability distribution from the previous iteration (the iteration before last gradient update). A PPO algorithm reduces the variance in the gradient, stabilizes training runs, and encourages the model to explore a wider region of the chemical space through the introduction of an entropy bonus term.

After the pretraining finished, we copied the weights to a separate model with identical architecture and trained with reinforcement learning using PPO. Because AutoDock Vina can predict different scores and poses for the same molecules due to random initiation, docking-derived reward terms for the same molecules sampled multiple times in one batch were averaged before the target functions were calculated for training stability. In each iteration after the molecules were sampled, model weights were updated by taking gradient steps on the target function through formula 18 using an Adam optimizer. In each iteration, all collected data were used for training the model for a maximum of 10 epochs. The trainer would continue into next iteration and collect new molecules for training if the K-L divergence between the latest predicted probability and the old probability exceeded 0.03.

The model was trained with RL until the mean entropy of the predicted probability of the tokens from the RNN started to decrease drastically. The training details including batch size, learning rate, hyperparameters α and ϵ, and the ratio between the reward terms λ for the 4 tasks are reported in Table S2.

## Data Availability

All the code for the iMiner reinforcement learning algorithm and workflow are provided in a private GitHub repository: https://github.com/THGLab/iMiner under reasonable requests.

## Supporting Information Available

Docking preparation and procedures, analysis of the generated molecules and additional model information (PDF)

## References

[ref1] Neves B. J.; Braga R. C.; Melo-Filho C. C.; Moreira-Filho J. T.; Muratov E. N.; Andrade C. H. QSAR-Based Virtual Screening: Advances and Applications in Drug Discovery. Front. Pharma 2018, 9, 1275 10.3389/fphar.2018.01275. PMC6262347 30524275

[ref2] Batool M.; Ahmad B.; Choi S. A Structure-Based Drug Discovery Paradigm. Int. J. Mol. Sci. 2019, 20, 2783 10.3390/ijms20112783. 31174387 PMC6601033

[ref3] Meng X. Y.; Zhang H. X.; Mezei M.; Cui M. Molecular docking: a powerful approach for structure-based drug discovery. Curr. Comput. Aided Drug Des. 2011, 7, 146–57. 10.2174/157340911795677602. 21534921 PMC3151162

[ref4] Amaro R. E.; Mulholland A. J. Multiscale methods in drug design bridge chemical and biological complexity in the search for cures. Nature Rev. Chem. 2018, 2, 0148 10.1038/s41570-018-0148. 30949587 PMC6445369

[ref5] Gaulton A.; Bellis L. J.; Bento A. P.; Chambers J.; Davies M.; Hersey A.; Light Y.; McGlinchey S.; Michalovich D.; Al-Lazikani B.; Overington J. P. ChEMBL: a large-scale bioactivity database for drug discovery. Nucleic Acids Res. 2012, 40, D1100–D1107. 10.1093/nar/gkr777. 21948594 PMC3245175

[ref6] Kim S.; Chen J.; Cheng T.; Gindulyte A.; He J.; He S.; Li Q.; Shoemaker B. A.; Thiessen P. A.; Yu B.; Zaslavsky L.; Zhang J.; Bolton E. E. PubChem in 2021: new data content and improved web interfaces. Nucleic Acids Res. 2021, 49, D1388–D1395. 10.1093/nar/gkaa971. 33151290 PMC7778930

[ref7] Sterling T.; Irwin J. J. ZINC 15 - Ligand Discovery for Everyone. J. Chem. Inform. Model. 2015, 55, 2324–2337. 10.1021/acs.jcim.5b00559. PMC4658288 26479676

[ref8] Grygorenko O. O.; Radchenko D. S.; Dziuba I.; Chuprina A.; Gubina K. E.; Moroz Y. S. Generating Multibillion Chemical Space of Readily Accessible Screening Compounds. iScience 2020, 23, 101873 10.1016/j.isci.2020.101873. 33145486 PMC7593547

[ref9] Polishchuk P. G.; Madzhidov T. I.; Varnek A. Estimation of the size of drug-like chemical space based on GDB-17 data. J. Comp.-Aid, Mol. Des. 2013, 27, 675–679. 10.1007/s10822-013-9672-4. 23963658

[ref10] Reulecke I.; Lange G.; Albrecht J.; Klein R.; Rarey M. Towards an integrated description of hydrogen bonding and dehydration: decreasing false positives in virtual screening with the HYDE scoring function. ChemMedChem: Chemistry Enabling Drug Discovery 2008, 3, 885–897. 10.1002/cmdc.200700319. 18384086

[ref11] Duffy B. C.; Zhu L.; Decornez H.; Kitchen D. B. Early phase drug discovery: cheminformatics and computational techniques in identifying lead series. Bioorg. Med. Chem. 2012, 20, 5324–5342. 10.1016/j.bmc.2012.04.062. 22938785

[ref12] Sanchez-Lengeling B.; Aspuru-Guzik A. Inverse molecular design using machine learning: Generative models for matter engineering. Science 2018, 361, 360–365. 10.1126/science.aat2663. 30049875

[ref13] Kusner M. J.; Paige B.; Hernández-Lobato J. M. Grammar variational autoencoder. Machine Learning. 2017, 1945–1954.

[ref14] Dai H.; Tian Y.; Dai B.; Skiena S.; Song L. Syntax-directed variational autoencoder for structured data. arXiv preprint 2018, arXiv:1802.08786 10.48550/arXiv.1802.08786.

[ref15] Subramanian A.; Saha U.; Sharma T.; Tailor N. K.; Satapathi S. Inverse Design of Potential Singlet Fission Molecules using a Transfer Learning Based Approach. arXiv preprint 2020, arXiv:2003.07666 10.48550/arXiv:2003.07666.

[ref16] Olivecrona M.; Blaschke T.; Engkvist O.; Chen H. Molecular de-novo design through deep reinforcement learning. J. Cheminform. 2017, 9, 48 10.1186/s13321-017-0235-x. 29086083 PMC5583141

[ref17] Popova M.; Isayev O.; Tropsha A. Deep reinforcement learning for de novo drug design. Sci. Adv. 2018, 4, eaap7885 10.1126/sciadv.aap7885. 30050984 PMC6059760

[ref18] Gottipati S. K.; Sattarov B.; Niu S.; Pathak Y.; Wei H.; Liu S.; Blackburn S.; Thomas K.; Coley C.; Tang J.; et al. Learning to navigate the synthetically accessible chemical space using reinforcement learning. Machine Learning. 2020, 3668–3679.

[ref19] Zhavoronkov A.; Ivanenkov Y. A.; Aliper A.; Veselov M. S.; Aladinskiy V. A.; Aladinskaya A. V.; Terentiev V. A.; Polykovskiy D. A.; Kuznetsov M. D.; Asadulaev A.; et al. Deep learning enables rapid identification of potent DDR1 kinase inhibitors. Nat. Biotechnol. 2019, 37, 1038–1040. 10.1038/s41587-019-0224-x. 31477924

[ref20] Zhavoronkov A.; Aladinskiy V.; Zhebrak A.; Zagribelnyy B.; Terentiev V.; Bezrukov D. S.; Polykovskiy D.; Shayakhmetov R.; Filimonov A.; Orekhov P.; et al. Potential 2019-nCoV 3C-like protease inhibitors designed using generative deep learning approaches. ChemRxiv 2020, 10.26434/chemrxiv.11829102.v2.

[ref21] Bung N.; Krishnan S. R.; Bulusu G.; Roy A. De novo design of new chemical entities for SARS-CoV-2 using artificial intelligence. Future Med. Chem. 2021, 13, 575–585. 10.4155/fmc-2020-0262. 33590764 PMC7888348

[ref22] Born J.; Manica M.; Cadow J.; Markert G.; Mill N. A.; Filipavicius M.; Janakarajan N.; Cardinale A.; Laino T.; Martínez M. R. Data-driven molecular design for discovery and synthesis of novel ligands: a case study on SARS-CoV-2. Mach. Learn.: Sci. Technol. 2021, 2, 025024 10.1088/2632-2153/abe808.

[ref23] Zhang J.; Chen H. De novo molecule design using molecular generative models constrained by ligand-protein interactions. J. Chem. Inf. Model. 2022, 62, 3291–3306. 10.1021/acs.jcim.2c00177. 35793555

[ref24] Li S.; Hu C.; Ke S.; Yang C.; Chen J.; Xiong Y.; Liu H.; Hong L. LS-MolGen: Ligand-and-Structure Dual-Driven Deep Reinforcement Learning for Target-Specific Molecular Generation Improves Binding Affinity and Novelty. J. Chem. Inf. Model. 2023, 63, 4207 10.1021/acs.jcim.3c00587. 37341350

[ref25] Jeon W.; Kim D. Autonomous molecule generation using reinforcement learning and docking to develop potential novel inhibitors. Sci. Rep. 2020, 10, 22104 10.1038/s41598-020-78537-2. 33328504 PMC7744578

[ref26] Luo S.; Guan J.; Jianzhu M.; et al. A 3D generative model for structure-based drug design. Advances in Neural Information Processing Systems. 2021, 6229–6239.

[ref27] Peng X.; Luo S.; Guan J.; Xie Q.; Peng J.; Ma J. Pocket2mol: Efficient molecular sampling based on 3d protein pockets. Machine Learning. 2022, 17644–17655.

[ref28] Qian H.; Lin C.; Zhao D.; et al. AlphaDrug: protein target specific de novo molecular generation. PNAS Nexus 2022, 1, pgac227 10.1093/pnasnexus/pgac227. 36714828 PMC9802440

[ref29] Zhang O.; Zhang J.; Jin J.; Zhang X.; Hu R.; Shen C.; Cao H.; Du H.; Kang Y.; Deng Y.; et al. ResGen is a pocket-aware 3D molecular generation model based on parallel multiscale modelling. Nature Machine Intelligence 2023, 5, 1020–1030. 10.1038/s42256-023-00712-7.

[ref30] Schneuing A.; Du Y.; Harris C.; Jamasb A.; Igashov I.; Du W.; Blundell T.; Lió P.; Gomes C.; Welling M.; et al. Structure-based drug design with equivariant diffusion models. arXiv preprint 2022, arXiv:2210.13695 10.48550/arXiv.2210.13695. PMC11659159 39653846

[ref31] Guan J.; Qian W.; Peng X. 3D equivariant diffusion for target-aware molecule generation and affinity prediction. Presented at the Eleventh International Conference on Learning Representations, Kigali, Rwanda, 2023.

[ref32] Krenn M.; Häse F.; Nigam A.; Friederich P.; Aspuru-Guzik A. Self-referencing embedded strings (SELFIES): A 100% robust molecular string representation. Mach. Learn.: Sci. Technol. 2020, 1, 045024 10.1088/2632-2153/aba947.

[ref33] Merity S.; Keskar N. S.; Socher R. Regularizing and Optimizing LSTM Language Models. arXiv preprint 2018, arXiv:1708.02182 10.48550/arXiv:1708.02182.

[ref34] Trott O.; Olson A. J. AutoDock Vina: Improving the speed and accuracy of docking with a new scoring function, efficient optimization, and multithreading. J. Comput. Chem. 2010, 31, 455–461. 10.1002/jcc.21334. 19499576 PMC3041641

[ref35] Lipinski C. A. Drug-like properties and the causes of poor solubility and poor permeability. J. Pharma. Tox. Meth. 2000, 44, 235–249. 10.1016/S1056-8719(00)00107-6. 11274893

[ref36] Dahlin J. L.; Nissink J. W. M.; Strasser J. M.; Francis S.; Higgins L.; Zhou H.; Zhang Z.; Walters M. A. PAINS in the assay: chemical mechanisms of assay interference and promiscuous enzymatic inhibition observed during a sulfhydryl-scavenging HTS. J. Med. Chem. 2015, 58, 2091–2113. 10.1021/jm5019093. 25634295 PMC4360378

[ref37] Jin Z.; Du X.; Xu Y.; Deng Y.; Liu M.; Zhao Y.; Zhang B.; Li X.; Zhang L.; Peng C.; et al. Structure of Mpro from SARS-CoV-2 and discovery of its inhibitors. Nature 2020, 582, 289–293. 10.1038/s41586-020-2223-y. 32272481

[ref38] Zhang C.-H.; Stone E. A.; Deshmukh M.; Ippolito J. A.; Ghahremanpour M. M.; Tirado-Rives J.; Spasov K. A.; Zhang S.; Takeo Y.; Kudalkar S. N.; et al. Potent noncovalent inhibitors of the main protease of SARS-CoV-2 from molecular sculpting of the drug perampanel guided by free energy perturbation calculations. ACS Cent. Sci. 2021, 7, 467–475. 10.1021/acscentsci.1c00039. 33786375 PMC7931627

[ref39] Cui W.; Yang K.; Yang H. Recent Progress in the Drug Development Targeting SARS-CoV-2 Main Protease as Treatment for COVID-19. Front. Mol. Biosci. 2020, 7, 398 10.3389/fmolb.2020.616341. PMC7746807 33344509

[ref40] Wildman S. A.; Crippen G. M. Prediction of physicochemical parameters by atomic contributions. J. Chem. Inform. Comp. Sci. 1999, 39, 868–873. 10.1021/ci990307l.

[ref41] Brenk R.; Schipani A.; James D.; Krasowski A.; Gilbert I. H.; Frearson J.; Wyatt P. G. Lessons learnt from assembling screening libraries for drug discovery for neglected diseases. ChemMedChem. 2008, 3, 435 10.1002/cmdc.200700139. 18064617 PMC2628535

[ref42] Tang S.; Chen R.; Lin M.; Lin Q.; Zhu Y.; Ding J.; Hu H.; Ling M.; Wu J. Accelerating autodock vina with gpus. Molecules 2022, 27, 3041 10.3390/molecules27093041. 35566391 PMC9103882

[ref43] Schulman J.; Wolski F.; Dhariwal P.; Radford A.; Klimov O. Proximal policy optimization algorithms. arXiv preprint 2017, arXiv:1707.06347 10.48550/arXiv.1707.06347.

[ref44] Everitt T.; Hutter M. Avoiding wireheading with value reinforcement learning. International Conference on Artificial General Intelligence 2016, 9782, 12–22. 10.1007/978-3-319-41649-6_2.

[ref45] Bickerton G. R.; Paolini G. V.; Besnard J.; Muresan S.; Hopkins A. L. Quantifying the chemical beauty of drugs. Nat. Chem. 2012, 4, 90–98. 10.1038/nchem.1243. 22270643 PMC3524573

[ref46] Daylight Chemical Information Systems, Inc. SMARTS - A Language for Describing Molecular Patterns. 1997; https://www.daylight.com/dayhtml_tutorials/languages/smarts/index.html.

[ref47] Degen J.; Wegscheid-Gerlach C.; Zaliani A.; Rarey M. On the Art of Compiling and Using’Drug-Like’Chemical Fragment Spaces. ChemMedChem: Chemistry Enabling Drug Discovery 2008, 3, 1503–1507. 10.1002/cmdc.200800178. 18792903

[ref48] Gobbi A.; Poppinger D. Genetic optimization of combinatorial libraries. Biotechnol. Bioeng. 1998, 61, 47–54. 10.1002/(SICI)1097-0290(199824)61:1<47::AID-BIT9>3.0.CO;2-Z. 10099495

[ref49] Landrum G. RDKit: Open-Source Cheminformatics Software; 2016.

[ref50] Salentin S.; Schreiber S.; Haupt V. J.; Adasme M. F.; Schroeder M. PLIP: fully automated protein-ligand interaction profiler. Nucleic Acids Res. 2015, 43, W443–7. 10.1093/nar/gkv315. 25873628 PMC4489249

[ref51] Lipinski C. A.; Lombardo F.; Dominy B. W.; Feeney P. J. Experimental and computational approaches to estimate solubility and permeability in drug discovery and development settings. Adv. Drug Delivery Rev. 1997, 23, 3–25. 10.1016/S0169-409X(96)00423-1. 11259830

[ref52] Daina A.; Michielin O.; Zoete V. SwissADME: a free web tool to evaluate pharmacokinetics, drug-likeness and medicinal chemistry friendliness of small molecules. Sci. Rep. 2017, 7, 42717 10.1038/srep42717. 28256516 PMC5335600

[ref53] Houston D. R.; Walkinshaw M. D. Consensus docking: improving the reliability of docking in a virtual screening context. J. Chem. Inform. Model. 2013, 53, 384–390. 10.1021/ci300399w. 23351099

[ref54] Jiang D.; Hsieh C.-Y.; Wu Z.; Kang Y.; Wang J.; Wang E.; Liao B.; Shen C.; Xu L.; Wu J.; et al. Interactiongraphnet: A novel and efficient deep graph representation learning framework for accurate protein-ligand interaction predictions. J. Med. Chem. 2021, 64, 18209–18232. 10.1021/acs.jmedchem.1c01830. 34878785

[ref55] Li J.; Guan X.; Zhang O.; Sun K.; Wang Y.; Bagni D.; Head-Gordon T. Leak Proof PDBBind: A Reorganized Dataset of Protein-Ligand Complexes for More Generalizable Binding Affinity Prediction. 2023, submitted to *The Journal of Physical Chemistry B*. 10.1021/acs.jpcb.5c08598 PMC13430955 41486605

[ref56] Guterres H.; Im W. Improving Protein-Ligand Docking Results with High-Throughput Molecular Dynamics Simulations. J. Chem. Inf. Model. 2020, 60, 2189–2198. 10.1021/acs.jcim.0c00057. 32227880 PMC7534544

[ref57] Selvaraj C.; Panwar U.; Dinesh D. C.; Boura E.; Singh P.; Dubey V. K.; Singh S. K. Microsecond MD Simulation and Multiple-Conformation Virtual Screening to Identify Potential Anti-COVID-19 Inhibitors Against SARS-CoV-2 Main Protease. Frontiers in Chemistry 2021, 8, 595273 10.3389/fchem.2020.595273. 33585398 PMC7873971

[ref58] Brown N.; Fiscato M.; Segler M. H.; Vaucher A. C. GuacaMol: benchmarking models for de novo molecular design. J. Chem. Inform. Model. 2019, 59, 1096–1108. 10.1021/acs.jcim.8b00839. 30887799

[ref59] Preuer K.; Renz P.; Unterthiner T.; Hochreiter S.; Klambauer G. Fréchet ChemNet distance: a metric for generative models for molecules in drug discovery. J. Chem. Inform. Model. 2018, 58, 1736–1741. 10.1021/acs.jcim.8b00234. 30118593

[ref60] Khan S. A.; Zia K.; Ashraf S.; Uddin R.; Ul-Haq Z. Identification of chymotrypsin-like protease inhibitors of SARS-CoV-2 via integrated computational approach. J. Biomol. Struct. Dyn. 2021, 39, 2607–2616. 10.1080/07391102.2020.1751298. 32238094

[ref61] Prabhakaran P.; Hebbani A. V.; Menon S. V.; Paital B.; Murmu S.; Kumar S.; Singh M. K.; Sahoo D. K.; Desai P. P. D. Insilico generation of novel ligands for the inhibition of SARS-CoV-2 main protease (3CLpro) using deep learning. Frontiers in Microbiology 2023, 14, 1194794 10.3389/fmicb.2023.1194794. 37448573 PMC10338188

[ref62] Erlanson D. A.; McDowell R. S.; O’Brien T. Fragment-based drug discovery. J. Med. Chem. 2004, 47, 3463–3482. 10.1021/jm040031v. 15214773

[ref63] Beigel J. H.; et al. Remdesivir for the Treatment of Covid-19 - Final Report. N. Engl. J. Med. 2020, 383, 1813–1826. 10.1056/NEJMoa2007764. 32445440 PMC7262788

[ref64] Unoh Y.; et al. Discovery of S-217622, a Noncovalent Oral SARS-CoV-2 3CL Protease Inhibitor Clinical Candidate for Treating COVID-19. J. Med. Chem. 2022, 65, 6499–6512. 10.1021/acs.jmedchem.2c00117. 35352927 PMC8982737

[ref65] El Khoury L.; Jing Z.; Cuzzolin A.; Deplano A.; Loco D.; Sattarov B.; Hédin F.; Wendeborn S.; Ho C.; El Ahdab D.; et al. Computationally driven discovery of SARS-CoV-2 M pro inhibitors: from design to experimental validation. Chemical science 2022, 13, 3674–3687. 10.1039/D1SC05892D. 35432906 PMC8966641

[ref66] Hegyi A.; Ziebuhr J. Conservation of substrate specificities among coronavirus main proteases. J. General Viro. 2002, 83, 595–599. 10.1099/0022-1317-83-3-595. 11842254

[ref67] Moghadasi S. A.; Heilmann E.; Khalil A. M.; Nnabuife C.; Kearns F. L.; Ye C.; Moraes S. N.; Costacurta F.; Esler M. A.; Aihara H.; von Laer D.; Martinez-Sobrido L.; Palzkill T.; Amaro R. E.; Harris R. S. Transmissible SARS-CoV-2 variants with resistance to clinical protease inhibitors. Sci. Adv. 2023, 9, eade8778 10.1126/sciadv.ade8778. 36989354 PMC10058310

[ref68] Ventola C. L. The antibiotic resistance crisis: part 1: causes and threats. P T 2015, 40, 277–83. 25859123 PMC4378521

[ref69] Polyak B. T.; Juditsky A. B. Acceleration of stochastic approximation by averaging. SIAM J. Contr. Opt. 1992, 30, 838–855. 10.1137/0330046.

[ref70] Wan L.; Zeiler M.; Zhang S.; Le Cun Y.; Fergus R. Regularization of neural networks using dropconnect. International conference on machine learning 2013, 1058–1066.

[ref71] Paszke A.; Gross S.; Chintala S.; Chanan G.; Yang E.; DeVito Z.; Lin Z.; Desmaison A.; Antiga L.; Lerer A. Automatic differentiation in PyTorch. Presented at the NIPS 2017 Workshop, 2017.

[ref72] Howard J.; Gugger S. Fastai: a layered API for deep learning. Information 2020, 11, 108 10.3390/info11020108.

[ref73] Kingma D. P.; Ba J. Adam: A method for stochastic optimization. arXiv preprint 2014, arXiv:1412.6980 10.48550/arXiv.1412.6980.

[ref74] Smith L. N. A disciplined approach to neural network hyper-parameters: Part 1-learning rate, batch size, momentum, and weight decay. arXiv preprint 2018, arXiv:1803.09820 10.48550/arXiv:1803.09820.

[ref75] Smith L. N.; Topin N. Super-convergence: Very fast training of neural networks using large learning rates. Artificial Intelligence and Machine Learning for Multi-Domain Operations Applications 2019, 1100612 10.1117/12.2520589.

[ref76] Williams R. J. Simple statistical gradient-following algorithms for connectionist reinforcement learning. Machine learning 1992, 8, 229–256. 10.1023/A:1022672621406.

