# Supplementary material for: Mining for Potent Inhibitors through Artificial Intelligence and Physics: A Unified Methodology for Ligand Based and Structure Based Drug Design
Source: J Chem Inf Model. 2024 Jun 6;64(24):9082–97. doi: 10.1021/acs.jcim.4c00634 (PMC11683870; doi:10.1021/acs.jcim.4c00634)
Supplement: Supplementary file 1 — ci4c00634_si_001.pdf [file ci4c00634_si_001.pdf]

Supplementary Information: Mining for Potent Inhibitors through Artificial Intelligence and Physics: A Unified Methodology for Ligand Based and Structure Based Drug Design

Jie Li*,† Oufan Zhang*,† Kunyang Sun*,† Yingze Wang,† Xingyi Guan,† Dorian Bagni,† Mojtaba Haghighatlari,† Fiona L. Kearns,‡ Conor Parks,‡ Rommie E. Amaro,‡ and Teresa Head-Gordon*,†,¶

†*Pitzer Center for Theoretical Chemistry, Department of Chemistry, University of California, Berkeley, CA, 94720*

‡*Department of Chemistry and Biochemistry University of California, San Diego, La Jolla, CA 92093-0340*

¶*Departments of Bioengineering and Chemical and Biomolecular Engineering, University of California, Berkeley, CA, USA*

E-mail: thg@berkeley.edu

Methodology Details

Tokens in the generative model. Here we provide a complete list of tokens used in the generative model:

- SELFIES tokens: 'C', '=C', 'Ring1', 'Branch1', 'N', '=Branch1', 'O', '=O', 'Ring2', 'Branch2', '=N', 'S', '#Branch1', '=Branch2', 'F', '#Branch2', 'C@H1', 'C@@H1', '#C', 'Cl', 'P',

'/C', 'NH1', '=Ring1', 'C@', 'C@@', 'O-1', 'Br', 'N+1', '#N', '\\C', '=Ring2', '/N', '=S', '=N+1', '\\N', 'I', '/O', '\\O', '\\S', 'S+1', '/S', '/C@@H1', '/C@H1', 'Se', '=N-1', '=P', 'N-1', '\\C@@H1', '\\C@H1', '/N+1', 'C-1', '#N+1', 'P+1', '\\NH1', 'OH0', '/Br', 'P@', 'P@@', '\\Cl', '\\O-1', '\\N+1', '/C@@', '-/Ring2', '/Cl', '/C@', 'PH1', '=Se'

- Functional tokens: 'Break'

When sampling molecules represented as SELFIES strings, the first token was always selected as the 'Break' token. Then each token was sampled with probability distribution predicted by the generative model. Once the 'Break' token was selected again, or the total number of tokens exceeded 500, a single molecule sampling process was considered complete.

Supervised fine-tuning of the network for specialized tasks. The same weight decay and dropout multiplier as in the pre-training with ChEMBL 24[1] were used in this stage. The pre-trained model was additionally tuned for 5-20 epochs to condition the molecule generation on the target substructures yet with structural diversity. For the task to generate analogs to the inhibitors reported by Zhang *et al.*,[2] the model was fine-tuned with 14 SMILES strings from PDB deposits (PDB ID 7L10, 7L11, 7L12, 7L13, 7L14, 7M8M, 7M8N, 7M8O, 7M8P, 7M8X, 7M8Y, 7M8Z, 7M90, 7M91). For the fragment growth design task, the fine-tuning set consisted of 867 molecules with substructure match from SciFinder, excluding the ones reported by Zhang *et al.*.

SMARTS patterns used in drug-likeliness reward function. Table S1 shows that ChEMBL has small percentages of cyclopentadiene and cyclopentadiene ylidenes, and of aromaticity breaking tautomers or saturation, which may risk their amplification in the generative/RL step of iMiner. To further exclude molecules that contain unstable or synthetically inaccessible functional groups, we incorporate the following undesirable SMARTS patterns into our drug-likeliness reward function to further constrain our chemical search space:

- "[C^2]1=[C^2]-[C^2]=[C^2]~[C;!d4]~[C;!^2;d2]1"
- "[C^2]1~[C^2]~[C^2]~[C^2]~[C;!^2;d2]~[N]1"
- "[#6^2]1~[#6^2]~[#6^3;!d4]~[#6^2]2~[#6^2]~[#6^2]~[#6^2]~[#6^2](~[*])~[#6^2]~2~[#6^2]~1"
- "[#6]1(=[*])[#6]=[#6][#6]=[#6]1"
- "[#6]1=[#6][R{2-}]=[R{2-}]1"
- "[#6^2]1~[#6^2]~[#6^2]~[#6^2]~[#6^1]~[#6^1]~1"
- "[#7,#8,#16]-[#9,#17,#35,#53]"
- "[r3,r4]@[r5,r6]"
- "[*]=[#6,#7,#8]=[*]"
- "[#7,#16]=[#16]"
- "[#8]-[#8]"

In addition to the patterns mentioned above, to ensure that we generate the correct pyrrole, we use the following SMARTS patterns to enforce our generated pyrrole to be correct:

- "[N^2]1~[C,N;^2](=[*])~[C,N;^2]~[C,N;^2]~[C^3]1"
- "[N^2]1~[C,N;^2]~[C,N;^2](=[*])~[C,N;^2]~[C;^3]1"
- ""[N^2]1~[C,N;^2]~[C,N;^2]~[C,N;^2](=[*])~[C;^3]1"
- "[C,N;^2](=[*])1~[N;^2]~[C,N;^2]~[C,N;^2]~[C;^3]1"
- "[C,N;^2]1~[N;^2]~[C,N;^2](=[*])~[C,N;^2]~[C;^3]1"
- "[C,N;^2]1~[N;^2]~[C,N;^2]~[C,N;^2](=[*])~[C;^3]1"

Mpro Active Site Receptor Preparation and Docking Procedures. For all four different generation tasks, we used SARS-CoV-2 Mpro conformation from the protein-ligand co-crystal structure (PDB ID 7L11[2]) as the receptor for molecular docking. After downloading the PDB file from Protein Data Bank,[3] we used PDBFixer from the OpenMM suite[4] to prepare the protein by fixing missing residues and removing water as well as other non-polymer entities. Then, we used the geometric center of the ligand (XF1) from the co-crystal structure [x=-22, y=-4, z=-28] as the center of the active site for receptor grid generation steps in AutoDock Vina.[5] AutoDockFR[6] was used to convert Mpro `.pdb` files to AutoDock Vina[5] compatible `.pdbqt` files. Additionally, Meeko and Open Babel[7] were used to convert generated molecule structure files to AutoDock Vina compatible `.pdbqt` files. Gastieger charges were used for all AutoDock Vina structures. A cubic receptor grid of 24Å x 24Å x 24Å was centered around the binding site's central coordinate (listed above), with a grid spacing of 1.0Å.

PDB Codes used for extracting the known binders of SARS-Cov-2 Mpro. In this study, we have borrowed a SARS-Cov-2 Mpro dataset from LP-PDBBind which consisted of 40 different non-covalent inhibitors manually extracted from published co-crystal structures on PDB.[2,8–19] This collection of molecules is referred to as "known binders" in this work. The PDB codes for these structures are shown here:

7VTH 7VU6 6W63 7L0D 6M2N 7AP6 7JU7 7L5D 7KX5 7L10 7L11 7L12 7L13 7L14 7S3S 7S3K 7S4B 7M8X 7M8M 7M8Z 7M8Y 7M8N 7M8O 7M90 7M91 7M8P 7N44 7EN8 7EN9 7LTJ 7RLS 7RM2 7RMB 7RME 7RMT 7RMZ 7RN4 7RNH 7RNK 8ACD 8ACL 7LME 7LMD 7LMF 7NT3

Supporting Tables

Table S1: Comparison of ChEMBL and after iMiner RL to eliminate undesirable molecules for different iMiner tasks. Here, we randomly sample 10,000 molecules from the ChEMBL dataset and our retrained model to compare their statistics of five different categories of undesirable chemical patterns. Such patterns are in the original ChEMBL dataset and hence our model only learns the distribution of such molecules from ChEMBL. These molecular features are further filtered out in the final set of molecules (for De Novo, Fragment, Position, Interaction iMiner tasks) when their drug likeliness scores are set to 0 when the corresponding SMARTS patterns occur during the training.

**Model**	ChEMBL	De Novo	Fragment	Position	Interaction
Unsaturated Benzene	0.55%	0.46%	0.95%	1.07%	0.65%
Unsaturated Naphthalene	0.02%	0.02%	0.14%	0.07%	0.01%
Wrong Pyrrole	0.29%	0.24%	0.0%	0.14%	0.19%
Cyclopentadiene Ylidene	0.96%	1.34%	0.59%	0.74%	0.86%
Benzyne	0.01%	0.0%	0.0%	0.0%	0.02%

Table S2: RL training details. The hyperparameters include batch size (bs), learning rate (lr), α and ϵ in the PPO loss function, and reward weights for Vina score λ_{vina}, drug-likeliness λ_{DL}, fragment similarity λ_{frag}, pharmacophore similarity λ_{phm} and interaction $\lambda_{interact}$.

**Task**	bs	lr	α	ϵ	λ_{vina}	λ_{DL}	λ_{frag}	λ_{phm}	$\lambda_{interact}$
**Unconditional *de novo* generation**	128	1e-6	0.04	0.08	3	1			
**Structural analog generation**	128	1e-6	0.03	0.08	2	1	4	2	
**Structure-based fragment growth**	128	1e-6	0.03	0.08	2	1	4		
**Interaction-based generation**	128	1e-6	0.03	0.08	2	1			5

Table S3: Comparison of the Frechet Chemnet Distances[20] between 1000 molecules randomly sampled from ChEMBL, 1000 molecules generated before RL optimization, 1000 molecules after RL optimization, and 40 known experimental binders extracted from LP-PDBBind.[21]

	**ChEMBL**	**Pre-RL**	**Post-RL**	**Known Binders**
**ChEMBL**	\	24.99	32.68	47.54
**Pre-RL**	\	\	3.80	15.44
**Post-RL**	\	\	\	11.25
**Known Binders**	\	\	\	\

Note: The Frechet Chemnet Distances implemented in Guacomol from Table 1 were run through some exponentials to fall from 0 to 1 as the other scores in their benchmark. Here, instead, we directly used the raw output to give a more direct representation of the distance, where a larger value means the two groups of smiles strings are more dissimilar and vice versa.

Table S4: Comparison of iMiner-unconstrained generation with various deep learning methods

**Methods**	**# of total mol.**	**QED**	**SA**	**LogP**	**# of Mpro mol.**	**Vina score**
AlphaDrug[22]	9617	0.42	0.80	5.65	98	−9.1
SBDD[23]	11,339	0.52	0.63	0.57	107	−4.5
Pocket2Mol[24]	13,647	0.58	0.78	1.40	103	−3.7
TargetDiff[25]	7118	0.50	0.58	1.63	82	−2.8
DiffSBDD[26]	9082	0.45	0.30	-0.10	76	−2.3
iMiner Unconstrained	12,557	0.38	2.55 #	5.25	125*	−8.9

Note: The table is borrowed from the DrugGen[27] Dataset, where they generate molecules with each method across various targets. The chemical properties reported (QED, SA, LogP, Vina Score) are all median values.
# This high SA score compared to other methods showed that iMiner could output more complicated molecules. In comparison, the average SA score for all the known ligands used in this work is 2.69.
* Since iMiner is explicitly optimized on Mpro, we calculated the median docking score for the final set of filtered molecules. The total median value of all generated molecules is -8.3.

Table S5: The filtered sets of molecules using iMiner structure-based analogs generation

Index	Canonical SMILES	newVina score	newIGN score
Structural Analogs			
1	C=CC(=O)n1cc2cc(-c3cc4ccc(OCC(F)F)nc4n(-c4ccc(C5CC5)nc4)c3=O)ccc2n1	-9.48	-9.90
2	C=CCNC(=O)c1ccc2cc(-c3ccc4nn(C)cc4c3)c(=O)n(-c3ccc(C4CC4)nc3)c2n1	-10.07	-8.91
3	CC1=C(COc2ccc(-n3c(=O)c(-c4ccc5nn(C)cc5c4)cc4ccc(OCC(F)(F)F)		

Table S5 (continued)

Index	Canonical SMILES	Vina score	IGN score
	nc43)cn2)CC=N1	-9.90	-8.63
4	CCOc1ccc2cc(-c3ccc4nn(C)cc4c3)c(=O)n(-c3ccc(C(F)(F)F)nc3)c2n1	-9.58	-8.67
5	COc1cc(C)ccc1-c1cc2ccc(NC(=O)C3CC3)nc2n(-c2ccc(C3CC3)nc2)c1=O	-10.03	-9.93
6	COc1ccc(-n2c(=O)c(-c3ccc4nn(C)cc4c3)cc3ccc(OC4CCC4)nc32)cn1	-9.60	-9.67
7	COc1ccc2cc(-c3cc4ccc(OC)nc4n(-c4ccc(C(F)(F)F)nc4)c3=O)ccc2n1	-10.03	-8.67
8	C[C@@H](NC(=O)c1cc(-c2ccc(F)cc2)c(=O)n(-c2ccc(Cl)nc2)c1)c1cc c(C(F)(F)F)nc1C1CC1	-10.11	-9.74
9	C[C@@H]1c2cnc(C(F)(F)F)nc2N1C(=O)c1cc(-c2ccc(F)cc2)c(=O)n(-c 2cccnc2)c1	-9.73	-9.17
10	C[C@H](NC(=O)c1cc(-c2ccc(F)cc2)c(=O)n(-c2cccnc2)c1)c1cnc(C(F) (F)F)nc1	-9.77	-8.94
11	Cc1c(S(=O)(=O)c2ccccc2F)cn(-c2cccnc2)c(=O)c1-c1ccc(F)cc1F	-9.59	-9.46
12	Cc1ccc(-n2c3c(cc(-c4cccc(Cl)c4Cl)c2=O)-c2ncccc2OC3)cn1	-10.10	-8.84
13	Cc1ccc(-n2c3c(cc(-c4cccc(F)c4C#N)c2=O)-c2ncccc2OC3)cn1	-10.09	-9.61
14	Cc1ccc(-n2c3c(cc(-c4cccc(F)c4Cl)c2=O)-c2ncccc2OC3)cn1	-10.25	-9.26
15	Cn1cc2cc(-c3cc4ccc(NCC(F)(F)F)nc4n(-c4ccc(C5CC5)nc4)c3=O)ccc2n1	-9.95	-9.03

Table S5 (continued)

Index	Canonical SMILES	Vina score	IGN score
16	Cn1cc2cc(-c3cc4ccc(OCC(F)F)nc4n(-c4ccc(C5CC5)nc4)c3=O)ccc2n1	-9.70	-9.47
17	N#Cc1c(Cl)cccc1-c1cc2c(n(-c3cncc(Cl)c3)c1=O)COc1cccnc1-2	-9.56	-9.04
18	N#Cc1c(F)ccc(F)c1-c1cc2c(n(-c3ccc(Cl)nc3)c1=O)COc1cccnc1-2	-9.95	-8.74
19	N#Cc1c(F)ccc(F)c1-c1cc2c(n(-c3cccnc3)c1=O)COc1cc(-n3ccc(-c4ccsc4)cc3=O)cnc1-2	-10.31	-9.63
20	N#Cc1c(F)cccc1-c1cc2c(n(-c3cccnc3)c1=O)COc1cc(-c3cncc(C(F)(F)F)c3)cnc1-2	-9.51	-9.25
21	N#Cc1c(F)cccc1-c1cc2c(n(-c3cccnc3)c1=O)COc1cc(F)cnc1-2	-10.35	-9.68
22	O=C(Cc1ccccc1)Nc1ncc2cc(-c3cccc(Cl)c3Cl)c(=O)n(-c3ccc(C4CC4)nc3)c2n1	-10.48	-10.40
23	O=c1c(-c2cccc3cccnc23)cc(-c2ccccn2)cn1-c1cnc2ccccc2c1	-10.93	-9.43

Table S6: The filtered sets of molecules using iMiner structure based fragment growth

Index	Canonical SMILES	newVina score	newIGN score
Structure-based Fragment Growth			
1	COc1cncc(-n2c3c(cc(-c4cccc(F)c4C#N)c2=O)-c2ncccc2OC3)c1	-9.95	-9.48
2	Cc1c(C(=O)N[C@@H](C)c2ccccc2)c2cccc(F)c2c(=O)n1-c1cccnc1		

Table S6 (continued)

Index	Canonical SMILES	Vina score	IGN score
		-9.35	-8.45
3	Cc1c(C(=O)N[C@H](c2ccc(F)c(F)c2)C2CCC2)c2cccc(F)c2c(=O)n1-c1ccc(F)nc1	-9.94	-10.59
4	Cc1c(C(=O)N[C@H](c2cccc(F)c2)C2CCC2)c2cc(F)cc(F)c2c(=O)n1-c1cncc(-c2ccnc(C3(C)CC3)c2)c1	-10.23	-10.82
5	Cc1ccc(-n2c(C)c(C(=O)N[C@H](c3cccc(F)c3)C3CC3)c3cccc(F)c3c2=O)cn1	-9.56	-9.58
6	Cc1ccc(-n2c3c(cc(-c4cccc(F)c4C#N)c2=O)-c2ncccc2OC3)cn1	-10.06	-9.17
7	Cc1ccncc1-n1c(C)c(C(=O)N[C@H](c2cccc(F)c2)C2CC2)c2cccc(F)c2c1=O	-9.72	-9.27
8	Cc1ccncc1-n1ccc2c(NC(=O)Cc3ccc(F)c(F)c3F)cccc2c1=O	-9.31	-8.98
9	O=c1c(-c2ccc(F)cc2)cc(S(=O)(=O)c2ccc(Cl)cc2)cn1-c1cccnc1	-9.38	-9.36
10	CCOc1cc2c(C(=O)N3CCCCC3)cn(-c3cccnc3)c(=O)c2cc1OCC	-8.14	-7.70
11	COc1ccc(-c2cc(-c3cccc(Cl)c3)c(=O)n(-c3cccnc3)c2)c(C#N)n1	-8.73	-8.06
12	COc1ccncc1-n1c(C)c(C(=O)N[C@H](c2cccc(F)c2)C2CC2)c2cccc(F)c2c1=O	-9.17	-9.30
13	Cc1c(Cl)cccc1-c1cc2c(n(-c3cccnc3)c1=O)COc1cccnc1-2	-9.49	-7.51
14	N#Cc1cc2cc3c(cc2n(-c2ccc(Cl)nc2)c1=O)OCO3	-8.06	-7.38

Table S6 (continued)

Index	Canonical SMILES	Vina score	IGN score
15	N#Cc1sccc1-c1cc(-c2ccccn2)cn(-c2ccc(Cl)nc2)c1=O	-8.42	-8.70
16	O=C1CCCc2c1cc(-c1nc(-c3ccc(Cl)cc3)cs1)c(=O)n2-c1cccnc1	-9.57	-7.51
17	O=c1c(-c2c[nH]c3ccccc23)cc(-c2ccccc2Cl)cn1-c1cccnc1	-9.21	-8.01
18	O=c1c(-c2ccc(Cl)cc2)cc(-c2ccc(Cl)cc2F)cn1-c1cccnc1	-8.79	-7.35
19	O=c1c(-c2ccc(F)cc2)cc(S(=O)(=O)c2ccc(Cl)cc2)cn1-c1cccnc1	-9.38	-9.36

Table S7: The filtered sets of molecules using iMiner ligand-protein interaction generation

Index	Canonical SMILES	newVina score	newIGN score
Interaction-based			
1	CC(=O)N1C2CCC1OC(c1cc3ncn(C)c(=O)c3c(N3CC[C@@H](CN)C3)c1C)C2	-9.14	-10.14
2	CC(C)C(c1c[nH]c2ccc(C(N)=O)cc12)c1nnc2c3ccccc3c(-c3ccccc3)nn12	-9.01	-9.24
3	CC1CC([C@](C)(NC(N)=S)c2cc(-c3ccccc3)cc(C(F)(F)F)c2)N(C(=O)N c2ccccc2C(F)(F)F)CS1	-9.14	-10.36
4	CC1CN(Cc2c(F)c(F)cc(F)c2F)c2nc(N3CCNC3)nc(-c3ccccc3Cl)c21	-9.04	-10.47
5	CNC(=O)c1cc(S(=O)(=O)N2CCN(C(=O)c3cccc(-c4ccccc4C)c3)CC2)ccc1O		

Table S7 (continued)

Index	Canonical SMILES	Vina score	IGN score
		-9.13	-8.97
6	COC1CCC2(CC1)Cc1ccc(-c3cc(C)cc(F)c3)cc1C21N=C(N)N(C2CC2)C1=O	-10.57	-8.87
7	COc1ccc(Cl)cc1C(=O)N[C@H](C)c1nc2c(C(F)(F)F)cccc2c(=O)n1-c1c cc(N)nc1	-9.33	-8.86
8	COc1cnc2c(Nc3ccc(F)c([C@]4(C)CS(=O)(=O)C(C)(C)C(=N)N4)c3)nc(N)nc2c1	-9.47	-9.27
9	C[C@@H]1Cn2c(nnc2C(F)(F)F)CN1C(=O)c1cccc(C(F)(F)c2n[nH]c(=O) c3ccccc23)c1	-9.83	-8.70
10	Cc1ccc(NS(=O)(=O)c2cccc(F)c2)cc1Nc1nc2ccccc2n1-c1cc(N)ncn1	-9.18	-8.88
11	Cn1c(=O)c2c(nc(N3CCC[C@@H(N)C3)n2Cc2ccccc2C#N)c2ccc(CC#N)cc21	-9.54	-9.24
12	N#C[C@H]1CCCC[C@@H]1n1nc(Nc2ccc(C(N)=O)cc2-c2cc(C(=O)NC3CCC3) cs2)c2c1CCCC2	-9.19	-11.29
13	NC1CCN(Cc2c(-c3ccc(F)cc3)nc3ccc(-c4ccccc4)cn23)CC1	-9.37	-8.67
14	N[C@]1(C(F)(F)F)C[C](c2cc(NC(=O)c3ncc(Cl)cc3F)cc(F)c2F)CNC1=O	-9.79	-8.89
15	O=C(CN1C(=O)[C@@]2(N[C@H]1Cc1ccccc1)C(=O)Nc1cc(Cl)ccc12)Nc1c ccc2[nH]c(-c3ccco3)nc12	-9.54	-9.25
16	O=C(NCCN1CCC2(CC1)C(=O)NCN2c1ccccc1)c1cc2cc(Cl)ccc2[nH]1	-9.04	-9.01
17	O=C(Nc1ccc2ccccc2c1C(=O)N1CCCC1)c1ccc(-c2nc3ccc(F)cc3c(=O)n2 -c2cccc(O)c2)cc1	-10.77	-9.58

Table S7 (continued)

Index	Canonical SMILES	Vina score	IGN score
18	O=C(c1cc(Cc2n[nH]c(=O)c3ccccc23)cc(F)c1F)N1CCN(c2ncccn2)CC1	-9.71	-8.86
19	O=C(c1ccc(-c2n[nH]c(=O)c3ccccc23)cc1)N1CCN(S(=O)(=O)c2ccc3ccccc3c2)CC1	-9.73	-9.49
20	O=C(c1cccc(Cc2n[nH]c(=O)c3c2CCCC3)c1)N1CCN(S(=O)(=O)C2CC2)CC1	-9.11	-10.94
21	O=C1C=C(c2c3n(c4ccccc24)Cc2cccc(CN4CCOCC4)c2C3=O)C(=O)N1	-9.72	-9.10
22	O=C1NCN(c2ccccc2)C12CCN(C(c1cc3ccccc3o1)c1nnnn1C1CCCC1)CC2	-10.30	-11.32
23	O=c1[nH]c(N2CCOCC2)nc(N[C@@H]2CCCNC2)c1-c1nc2ccccc2s1	-9.02	-9.63

Supporting Figures

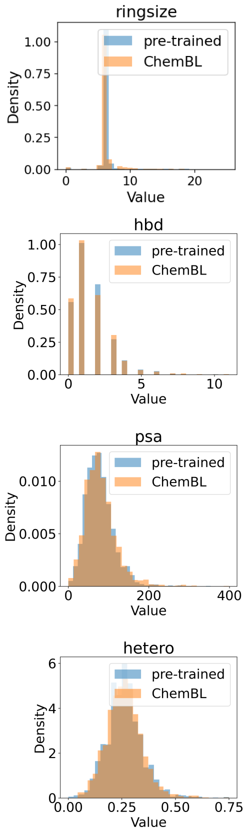

Figure S1: *Distribution comparisons for 13 different properties of the generated molecules from the pretrained model with molecules from the training dataset (ChEMBL).* The molecular properties considered are well-recognized chemical features related to the drug-likeliness of a molecule which can be obtained through 2D topological connectivity of the molecule: the size of the largest ring in the molecule (ringsize), molecular weight (mw), approximate log partition coefficient between octanol and water (alogP),[28] number of hydrogen bond acceptors (hba) and donors (hbd), number of rotatable bonds (rotb), number of aliphatic (alip) and aromatic rings (arom), polarizable surface area (PSA), number of structural alerts (alerts),[29] fraction of sp^3 hybridized carbons (fracsp3), number of heavy atoms (heavy) and fraction of non-carbon atoms in all heavy atoms (hetero).

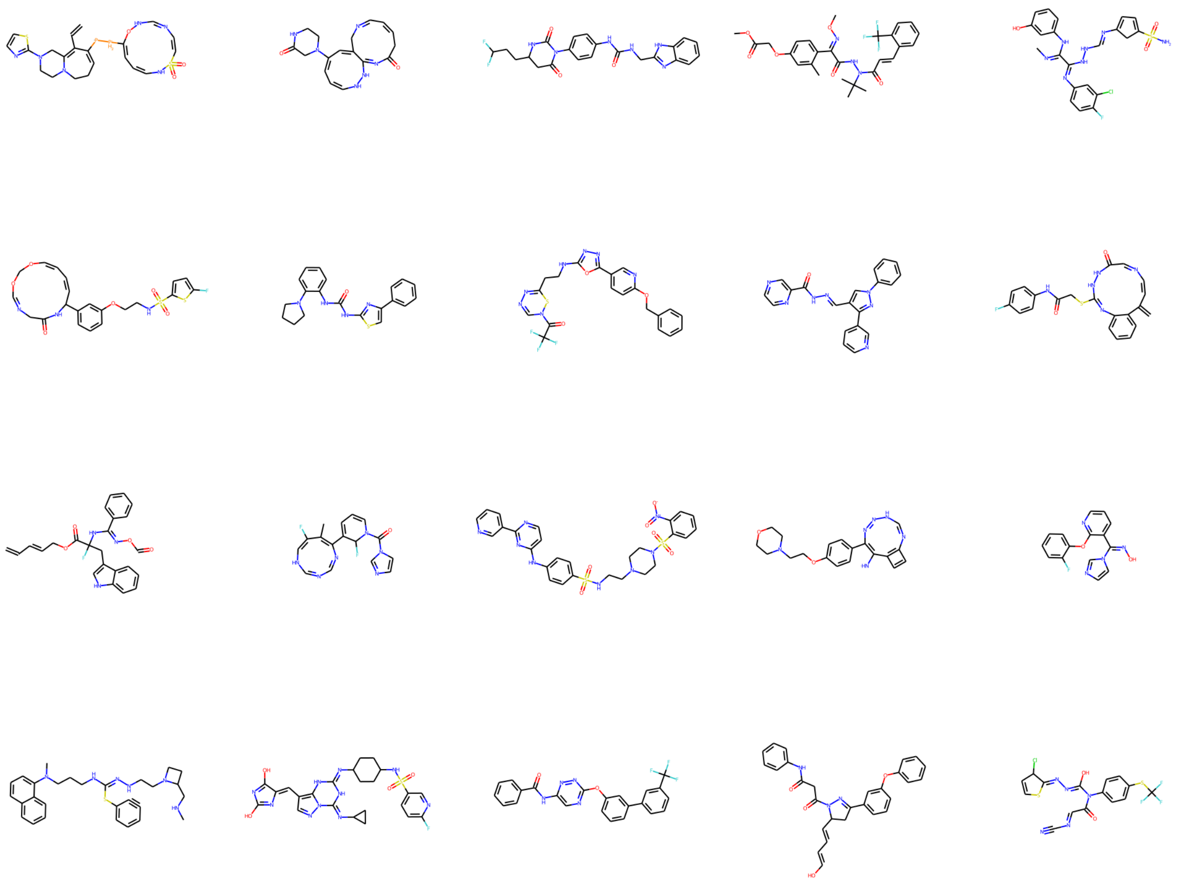

Figure S2: Example molecules generated using reinforcement learning without utilizing the drug-likeliness metric as an additional reward. Many of these molecules are not drug-like, i.e. having large rings, or having a high proportion of hetero atoms.

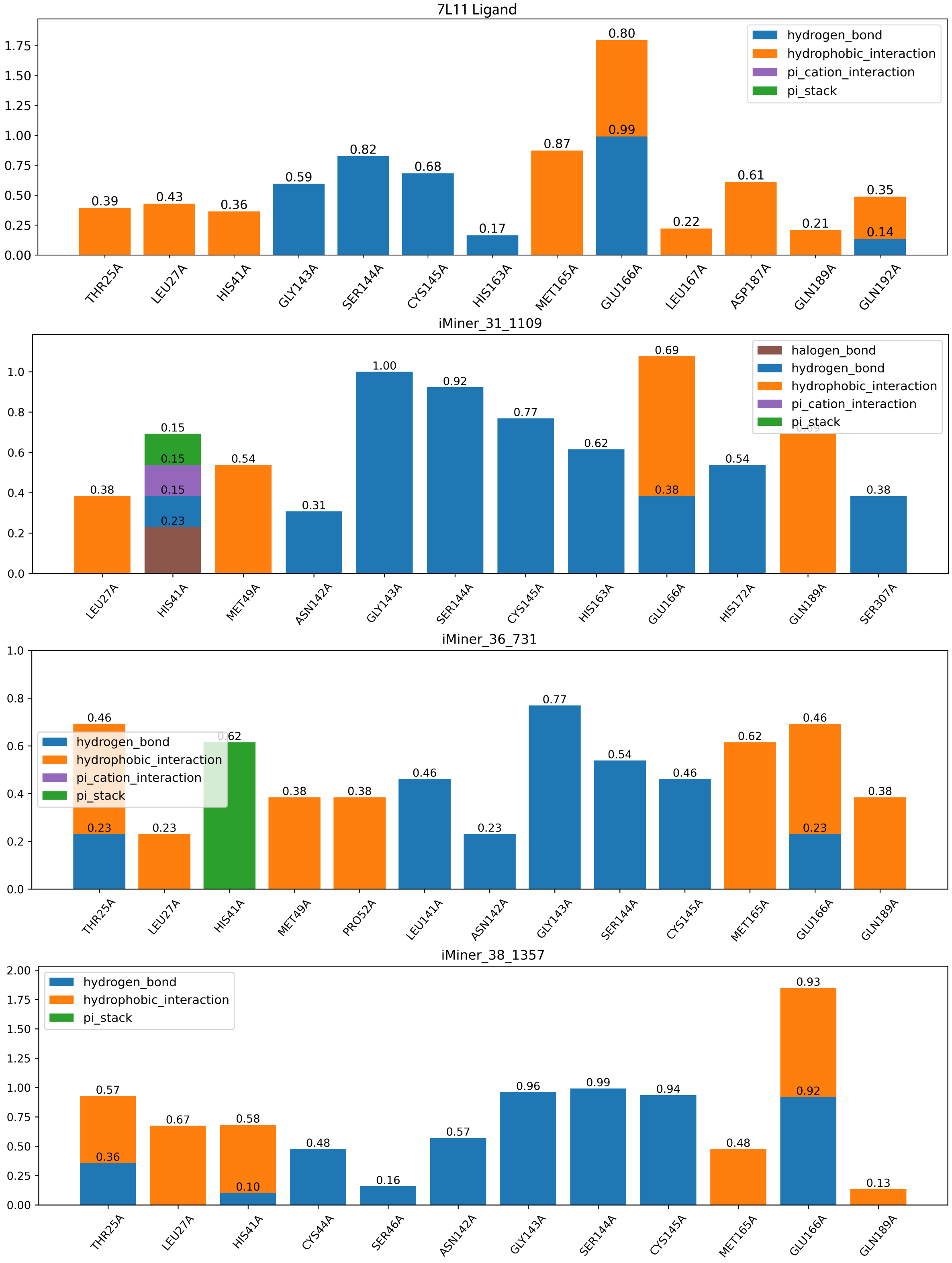

Figure S3: *The residue-level protein-ligand interaction frequency plots across different ligands.* 7L11 ligand is the native ligand from its PDB structure; iMiner_31_1109 was proposed from iMiner fragment growth; and iMiner_36_731 and iMiner_38_1357 were both proposed from iMiner interaction-based design.

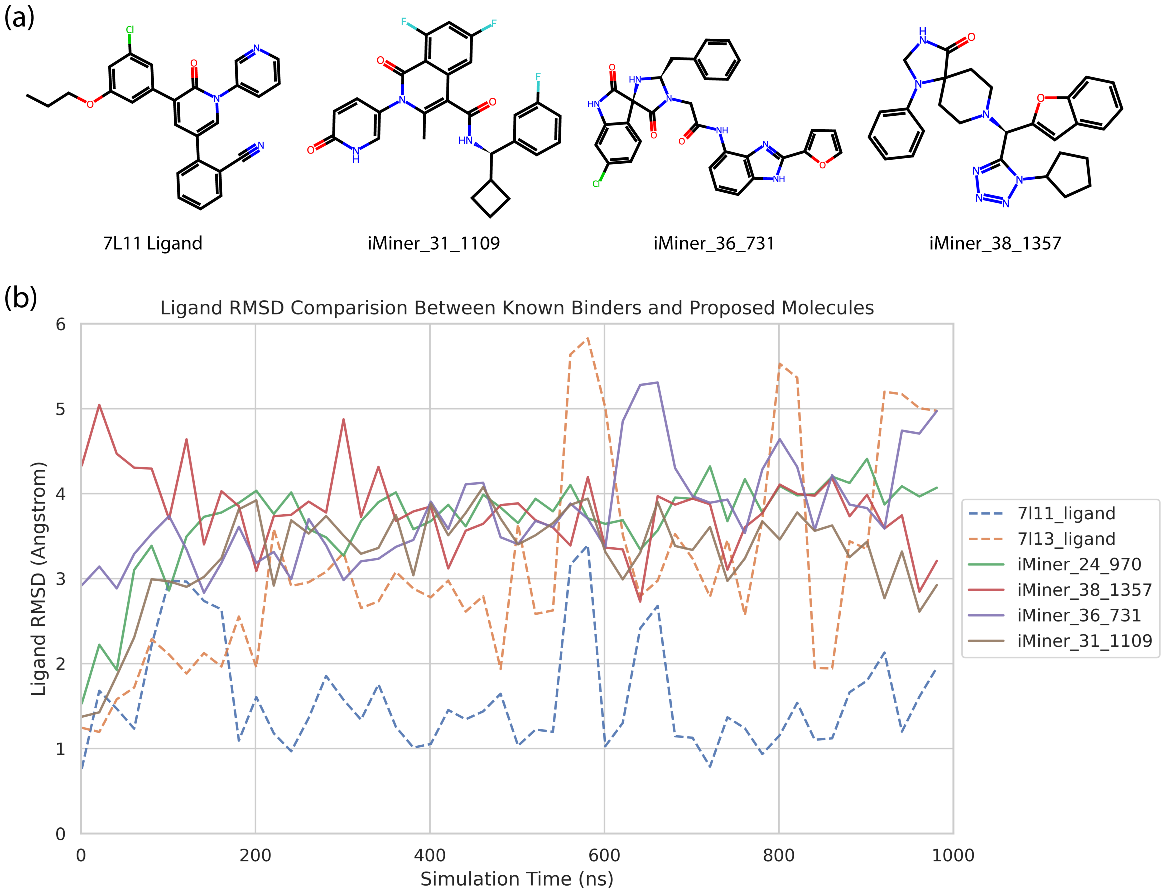

Figure S4: *Ligand RMSD analysis from long MD simulations.* a). 2D visualization of the other best molecules generated based on different iMiner protocols. b). The ligand RMSD analysis of the 4 proposed molecules and 2 known binders (PDB code: 7L11 and 7L13) over 1 μs simulations.

References

(1) Gaulton, A.; Bellis, L. J.; Bento, A. P.; Chambers, J.; Davies, M.; Hersey, A.; Light, Y.; McGlinchey, S.; Michalovich, D.; Al-Lazikani, B.; Overington, J. P. ChEMBL: a large-scale bioactivity database for drug discovery. *Nuc. Acids Res.* **2012**, *40*, D1100–D1107.

(2) Zhang, C.-H. et al. Potent noncovalent inhibitors of the main protease of SARS-CoV-2 from molecular sculpting of the drug perampanel guided by free energy perturbation calculations. *ACS Cent. Sci.* **2021**, *7*, 467–475.

(3) Berman, H.; Westbrook, J.; Feng, Z.; Gilliland, G.; Bhat, T.; Weissig, H.; Shindyalov, I.; Bourne, P. The Protein Data Bank. *Nucleic Acids Research* **2000**, *28*, 235–242.

(4) Eastman, P. et al. OpenMM 4: A Reusable, Extensible, Hardware Independent Library for High Performance Molecular Simulation. *Journal of Chemical Theory and Computation* **2013**, *9*, 461–469.

(5) Trott, O.; Olson, A. J. AutoDock Vina: Improving the speed and accuracy of docking with a new scoring function, efficient optimization, and multithreading. *J. Comp. Chem.* **2010**, *31*, 455–461.

(6) Ravindranath, P. A.; Forli, S.; Goodsell, D. S.; Olson, A. J.; Sanner, M. F. AutoDockFR: advances in protein-ligand docking with explicitly specified binding site flexibility. *PLoS Comp. Bio.* **2015**, *11*, e1004586.

(7) The Open Babel Package. 2016; `http://openbabel.org/`.

(8) Unoh, Y.; Uehara, S.; Nakahara, K.; Nobori, H.; Yamatsu, Y.; Yamamoto, S.; Maruyama, Y.; Taoda, Y.; Kasamatsu, K.; Suto, T.; others Discovery of S-217622, a noncovalent oral SARS-CoV-2 3CL protease inhibitor clinical candidate for treating COVID-19. *J. Med. Chem.* **2022**, *65*, 6499–6512.

(9) Lockbaum, G. J.; Reyes, A. C.; Lee, J. M.; Tilvawala, R.; Nalivaika, E. A.; Ali, A.; Kurt Yilmaz, N.; Thompson, P. R.; Schiffer, C. A. Crystal structure of SARS-CoV-2 main protease in complex with non-covalent inhibitor ML188. *Viruses* **2021**, *13*, 174.

(10) Su, H.-x.; Yao, S.; Zhao, W.-f.; Li, M.-j.; Liu, J.; Shang, W.-j.; Xie, H.; Ke, C.-q.; Hu, H.-c.; Gao, M.-n.; others Anti-SARS-CoV-2 activities in vitro of Shuanghuanglian preparations and bioactive ingredients. *Acta Pharm. Sin.* **2020**, *41*, 1167–1177.

(11) Günther, S.; Reinke, P. Y.; Fernández-García, Y.; Lieske, J.; Lane, T. J.; Ginn, H. M.; Koua, F. H.; Ehrt, C.; Ewert, W.; Oberthuer, D.; others X-ray screening identifies active site and allosteric inhibitors of SARS-CoV-2 MPro. *Science* **2021**, *372*, 642–646.

(12) Drayman, N.; DeMarco, J. K.; Jones, K. A.; Azizi, S.-A.; Froggatt, H. M.; Tan, K.; Maltseva, N. I.; Chen, S.; Nicolaescu, V.; Dvorkin, S.; others Masitinib is a broad coronavirus 3CL inhibitor that blocks replication of SARS-CoV-2. *Science* **2021**, *373*, 931–936.

(13) Kitamura, N.; Sacco, M. D.; Ma, C.; Hu, Y.; Townsend, J. A.; Meng, X.; Zhang, F.; Zhang, X.; Ba, M.; Szeto, T.; others Expedited approach toward rational design of noncovalent SARS-CoV-2 MPro inhibitors. *J. Med. Chem.* **2021**, *65*, 2848–2865.

(14) Glaser, J.; Sedova, A.; Galanie, S.; Kneller, D. W.; Davidson, R. B.; Maradzike, E.; Del Galdo, S.; Labbé, A.; Hsu, D. J.; Agarwal, R.; others Hit expansion of a noncovalent SARS-CoV-2 main protease inhibitor. *ACS Pharm. Trans. Sci.* **2022**, *5*, 255–265.

(15) Deshmukh, M. G.; Ippolito, J. A.; Zhang, C.-H.; Stone, E. A.; Reilly, R. A.; Miller, S. J.; Jorgensen, W. L.; Anderson, K. S. Structure-guided design of perampanel-derived pharmacophore targeting SARS-CoV-2 main protease. *Structure* **2021**, *29*, 823–833.

(16) Hou, N.; Shuai, L.; Zhang, L.; Xie, X.; Tang, K.; Zhu, Y.; Yu, Y.; Zhang, W.; Tan, Q.; Zhong, G.; others Development of highly potent noncovalent inhibitors of SARS-CoV-2 3CL-pro. *ACS central science* **2023**, *9*, 217–227.

(17) Kneller, D. W.; Li, H.; Galanie, S.; Phillips, G.; Labbé, A.; Weiss, K. L.; Zhang, Q.; Arnould, M. A.; Clyde, A.; Ma, H.; others Structural, electronic, and electrostatic determinants for inhibitor binding to subsites S1 and S2 in SARS-CoV-2 main protease. *J. Med. Chem.* **2021**, *64*, 17366–17383.

(18) Gao, S.; Sylvester, K.; Song, L.; Claff, T.; Jing, L.; Woodson, M.; Weisse, R. H.; Cheng, Y.; Schaakel, L.; Petry, M.; others Discovery and crystallographic studies of trisubstituted piperazine derivatives as non-covalent SARS-CoV-2 MPro inhibitors with high target specificity and low toxicity. *J. Med. Chem.* **2022**, *65*, 13343–13364.

(19) Han, S. H.; Goins, C. M.; Arya, T.; Shin, W.-J.; Maw, J.; Hooper, A.; Sonawane, D. P.; Porter, M. R.; Bannister, B. E.; Crouch, R. D.; others Structure-based optimization of ML300-derived, noncovalent inhibitors targeting the severe acute respiratory syndrome coronavirus 3CL protease (SARS-CoV-2 3CLpro). *J. Med. Chem.* **2021**, *65*, 2880–2904.

(20) Preuer, K.; Renz, P.; Unterthiner, T.; Hochreiter, S.; Klambauer, G. Fréchet ChemNet distance: a metric for generative models for molecules in drug discovery. *J. Chem. Inform. Model.* **2018**, *58*, 1736–1741.

(21) Li, J.; Guan, X.; Zhang, O.; Sun, K.; Wang, Y.; Bagni, D.; Head-Gordon, T. Leak Proof PDB-Bind: A Reorganized Dataset of Protein-Ligand Complexes for More Generalizable Binding Affinity Prediction. *under review* **2023**,

(22) Qian, H.; Lin, C.; Zhao, D.; Xu, L. AlphaDrug: protein target specific de novo molecular generation. *PNAS Nexus* **2022**, *1*, pgac227–238.

(23) Luo, S.; Guan, J.; Ma, J.; Peng, J. A 3D generative model for structure-based drug design. Advances in Neural Information Processing Systems. 2021; pp 6229–6239.

(24) Peng, X.; Luo, S.; Guan, J.; Xie, Q.; Peng, J.; Ma, J. Pocket2mol: Efficient molecular sampling based on 3d protein pockets. International Conference on Machine Learning. 2022; pp 17644–17655.

(25) Guan, J.; Qian, W. W.; Peng, X.; Su, Y.; Peng, J.; Ma, J. 3D equivariant diffusion for target-aware molecule generation and affinity prediction. The Eleventh International Conference on Learning Representations. Kigali, Rwanda, 2023.

(26) Schneuing, A.; Du, Y.; Harris, C.; Jamasb, A.; Igashov, I.; Du, W.; Blundell, T.; Lió, P.; Gomes, C.; Welling, M.; others Structure-based drug design with equivariant diffusion models. *arXiv preprint arXiv:2210.13695* **2022**,

(27) Qian, H.; Zhou, J.; Tu, S.; Xu, L. DrugGen: a database of de novo-generated molecular binders for specified target proteins. *Database* **2023**, *2023*, baad090.

(28) Wildman, S. A.; Crippen, G. M. Prediction of physicochemical parameters by atomic contributions. *J. Chem. Inform. Comp. Sci.* **1999**, *39*, 868–873.

(29) Brenk, R.; Schipani, A.; James, D.; Krasowski, A.; Gilbert, I. H.; Frearson, J.; Wyatt, P. G. Lessons learnt from assembling screening libraries for drug discovery for neglected diseases. *ChemMedChem* **2008**, *3*, 435.
